# ALDH1A3 promotes aggressive basal-like pancreatic cancer through an AP-1/RUNX2 enhancer network

**DOI:** 10.1038/s41388-025-03530-w

**Published:** 2025-08-08

**Authors:** Xiaoping Zou, Shuang Nie, Jing Cao, Mengyue Shi, Kathleen Schuck, Zhao Shi, Lingling Zhang, Hongzhen Li, Yifeng Sun, Chao Fang, Jingxiong Hu, Yiqi Niu, Yuanyuan Yu, Zhiheng Zhang, Chao Li, Mingyue Hu, Lei Wang, Kuirong Jiang, Zipeng Lu, Jan Akkan, Susanne Raulefs, Christoph Kahlert, Susanne Roth, Ingrid Herr, Yuan Wan, Andre Mihaljevic, Xuetian Qian, Qi Zhang, Maggie Haitian Wang, Jörg Kleeff, Helmut Friess, Zuguang Gu, Christoph W. Michalski, Shanshan Shen, Bo Kong

**Affiliations:** 1 Department of Gastroenterology, Affiliated Drum Tower Hospital of Nanjing University, Medical School, Nanjing, China; 2 Department of General, Visceral and Transplantation Surgery, University of Heidelberg, Heidelberg, Germany; 3 Department of Surgery, Klinikum rechts der Isar, School of Medicine, Technical University of Munich (TUM), Munich, Germany; 4 Department of General and Visceral Surgery, Ulm University Hospital, Ulm, Germany; 5 Department of General Surgery, Union Medical Center of Tianjin, Nankai University, Tianjin, China; 6 Pancreas Center, The First Affiliated Hospital of Nanjing Medical University, Nanjing, China; 7 The Pq Laboratory of Micro/Nano BiomeDx/Rx, Department of Biomedical Engineering, Binghamton University, State University of New York, Binghamton, NY USA; 8 Department of General, Visceral and Transplant Surgery, University Hospital Tübingen, Tübingen, Germany; 9 Department of Hepatobiliary and Pancreatic Surgery, the First Affiliated Hospital, Zhejiang, University School of Medicine, Hangzhou, China; 10 Centre for Clinical Research and Biostatistics (CCRB), Chinese University of Hong Kong, Hong Kong, China; 11 Department of Visceral, Vascular and Endocrine Surgery, Martin Luther University Halle-Wittenberg, Halle, Germany; 12 Bioinformatics and Omics Data Analytics, DKFZ, Heidelberg, Germany

**Keywords:** Metastasis, Oncogenes

## Abstract

The basal-like transcriptional subtype of pancreatic ductal adenocarcinoma (PDAC) is linked to therapy resistance and poor prognosis. The cancer stem cell marker aldehyde dehydrogenase 1A3 (ALDH1A3) is a critical enzyme in acetaldehyde metabolism, but the interconnection to the basal-like subtype is poorly understood. Here, we identified ALDH1A3 as a key gene, which correlates with reduced survival and increased tumor growth. Functional studies revealed interaction of ALDH1A3 with genes like FAM3C, MCC, PMEPA1, and IRS2, forming a network driving PDAC progression. Chromatin profiling showed that ALDH1A3 affects acetylation of histone 3, mediating AP-1 activity, particularly via FOS family members, activating oncogenic pathways such as MAPK and TNF signaling. RUNX2 emerged as a therapeutic target within this network, as its knockdown disrupted MAPK signaling and reduced tumor growth. These findings emphasize the role of ALDH1A3 in linking nuclear metabolic-epigenetic programming in basal-like PDAC, highlighting it as a promising therapeutic target for novel treatment strategies.

## Introduction

Pancreatic ductal adenocarcinoma (PDAC) is a deadly malignancy, notorious for its low 5-year survival rate and typically late diagnosis [1]. PDAC is histologically diverse, with transcriptional subtypes influencing its progression, treatment responses, and overall prognosis [2–6]. Advanced RNA profiling has identified two primary molecular subtypes: the classical epithelial subtype, also known as the progenitor subtype, and the more aggressive basal-like subtype. The latter is also referred to as quasi-mesenchymal or squamous, often showing poorer outcomes following treatment.

We recently identified a specific aggressive PDAC subtype marked by elevated expression of the cancer stem cell marker aldehyde dehydrogenase 1 family member A3 (ALDH1A3), which is associated with adverse prognosis in various cancers [7, 8]. ALDH1A3 participates in critical metabolic processes, such as the conversion of acetaldehyde to acetate, leading to the production of acetyl-coenzyme A (A-CoA), a molecule involved in histone H3 lysine 27 (H3K27) acetylation in smooth muscle cells. This action specifically reshapes the enhancer architecture of SMCs, thereby coordinating the function of ALDH1A3 in promoting cellular proliferation and glycolysis [7, 9].

Furthermore, the transcription factor (TF) AP-1, comprising subunits from the JUN and FOS families [10], plays a pivotal role in PDAC subtype differentiation through its involvement in KRAS-mediated oncogenic processes and tumor necrosis factor signaling [11–13]. Notably, cJUN/AP-1 activation and its affiliated enhancer networks have been linked to the basal-like subtype, whereas the JUNB/AP-1-dependent enhancer network is predominant in the classical subtype [14]. In particular, cJUN/AP-1-mediated basal-like differentiation is coupled with intrinsic activation of tumor necrosis factor (TNF)-α within PDAC cells. Stroma-derived TNF-α, primarily from macrophages, seems to play an important role in this process; however, the cell-intrinsic mechanisms involved in the differentiation process remain elusive.

Here, we demonstrate that PDAC tumors with high ALDH1A3 expression significantly overlap with the basal-like subtype, as detected by the use of various cell lines, mouse models, and human tissues. Mechanistically, ALDH1A3 promotes an oncogenic, basal-like-specific transcriptional program by regulating the FOSL2/AP-1-mediated enhancer network, converging in oncogenic mitogen-activated protein kinase (MAPK) and TNF signaling. Using an integrative multi-omics approach, we identified the runt-related transcription factor 2 (RUNX2) as a druggable target for the downregulation of ALDH1A3, which serves as an intrinsic factor facilitating basal-like transcriptional differentiation.

## Methods

### Patient material and tissue collection

PDAC tissues were obtained for immunohistochemical analysis from patients who underwent pancreatic resections. All sample diagnoses were histologically confirmed. Samples were either snap-frozen in liquid nitrogen or fixed in paraformaldehyde solution for 24 h, and subsequently paraffin-embedded for histological analysis. Detailed clinical and pathological data were collected from each patient. Patient samples for RNA-sequencing (RNA-seq) were obtained from the First Affiliated Hospital of Nanjing Medical University and the Affiliated Drum Tower Hospital of Nanjing University.

### Mouse lines

Mice containing two floxed alleles of Aldh1a3 were obtained from the Institute of Genetics, Molecular and Cellular Biology, France (IGBMC). The exon 8-9 of the Aldh1a3 allele on chromosome 7 in mice is flanked by two loxP sites, as previously described [15, 16]. The Loxp-STOP-Lox-Kras^G12D/+^ (LSL-Kras^G12D/+^; 008179) and Ptf1α^CreER™/+^ (19378) mutant mouse lines were obtained from the Jackson Laboratory, and the pancreas-specific Cre recombinase line Ptf1a^Cre/+^ (also known as p48^Cre/+^) was obtained from our previous studies [17, 18]. Wild type (WT or C57BL/6 J) and BALB/c nu/nu athymic mice were obtained from Charles River. The Rosa26 conditional knock-in line was generated using a custom service provided by Cyagen Biosciences Inc. Correctly targeted ES clones were confirmed and selected for blastocyst microinjection, followed by chimera production. Germline transmission was confirmed in founders via crossbreeding with the wild types.

### Human PDAC cell lines

The human PDAC cell lines used in this study, AsPC-1, HPAC, and PANC-1, were purchased from the ATCC. All cell lines were cultured in the recommended medium according to ATCC protocols, supplemented with 10% (v/v) fetal bovine serum (FBS), and maintained in a humidified incubator at 37 °C with 5% CO_2_.

### Analysis of data from the cancer genome atlas and compass trial

Level 3 expression data derived from the Cancer Genome Atlas (TCGA) and clinical data were downloaded from the UCSC Xena data portal (https://xena.ucsc.edu/). Compass trial data were obtained from EGA under the accession code EGAS00001002543 (for advanced tumors) and from the International Cancer Genome Consortium (ICGC) data portal (for resected primary tumors). For the compass trial data, reads were aligned to the human reference genome (hg38) and transcriptome (Ensembl v84) using HISAT [19] and Bowtie2 [20], and the expression levels of transcripts were calculated using RSEM [21–23].

### ALDH1A3 network score and analysis across datasets

The ALDH1A3 score was calculated using eight previously described genes. For each gene, samples with the top 50% expression value were given a score of +1, while samples with the bottom 50% expression value were given a score of –1. Coefficients were assigned to each gene based on their interaction. There were 13 lines representing relationship among 8 genes. The double-sided arrow occupied 1, and the single-sided arrow occupied 0.5. The specific coefficients for each gene were as follows:

$${\rm{ALDH}}1{\rm{A}}3:3/13* 100 \% =23.08 \%$$

$${\rm{FAM}}3{\rm{C}}:2.5/13* 100 \% =19.23 \%$$

$${\rm{EMP}}1:1.5/13* 100 \% =11.54 \%$$

$${\rm{PMEPA}}1:2/13* 100 \% =15.38 \%$$

$${\rm{MCC}}:1.5/138100 \% =11.54 \%$$

$${\rm{IRS}}2:1/13* 100 \% =7.69 \%$$

$${\rm{MAML}}2:1/13* 100 \% =7.69 \%$$

$${\rm{SP}}100:0.5/13* 100 \% =3.85 \%$$

The ALDH1A3 network score for each sample was the sum of the scores for the eight genes multiplied by their respective coefficients. The patients were divided into high- and low-score groups according to the median ALDH1A3-network score level. Samples were classified into different subtypes based on the results of Moffitt et al. [3] and Collisson et. al. [2] and Bailey et al. [4] NMF was used to produce two clusters using 50 (48 with a unique match in our data) tumor-specific transcripts from Moffitt et al., three clusters using 62 (61 with a unique match in our data) transcripts identified by Collisson et al. [2] and four clusters using 613 (463 with a unique match in our data) differentially expressed transcripts from Bailey et al. [4] A heatmap was generated using MORPHEUS (https://software.broadinstitute.org/morpheus/).

### Statistical analysis

For animal studies, a minimum of 4 mice were used in this study. No animal was excluded from the analysis. Random Number Table method was used to determine how animals were allocated to experimental groups. The investigator was not blinded to the treatment. Statistical analyses were performed using either GraphPad Prism V.7 (GraphPad) or IBM SPSS V.20 (Statistical Package for the Social Sciences, IBM). The variance is similar between the groups that are being statistically compared. Chi-square (χ2) or Fisher’s exact tests were used to compare the distributions of categorical factors among the various groups. Pearson correlation coefficients were used for correlation analysis. All experiments were repeated at least three times, with the exception of the CUT&Tag and ATAC-seq assays, which were conducted twice. If not otherwise mentioned, an unpaired Student’s t test was employed for two-group comparisons. The threshold for statistical significance was set at *p* < 0.05. Unless otherwise indicated, results are expressed as the mean ± SD. For every figure, statistical tests are justified as appropriate. And the data meet the assumptions of the tests.

## Results

### Association of ALDH1A3 expression with the basal-like aggressive PDAC subtype

**Fig. 1:**
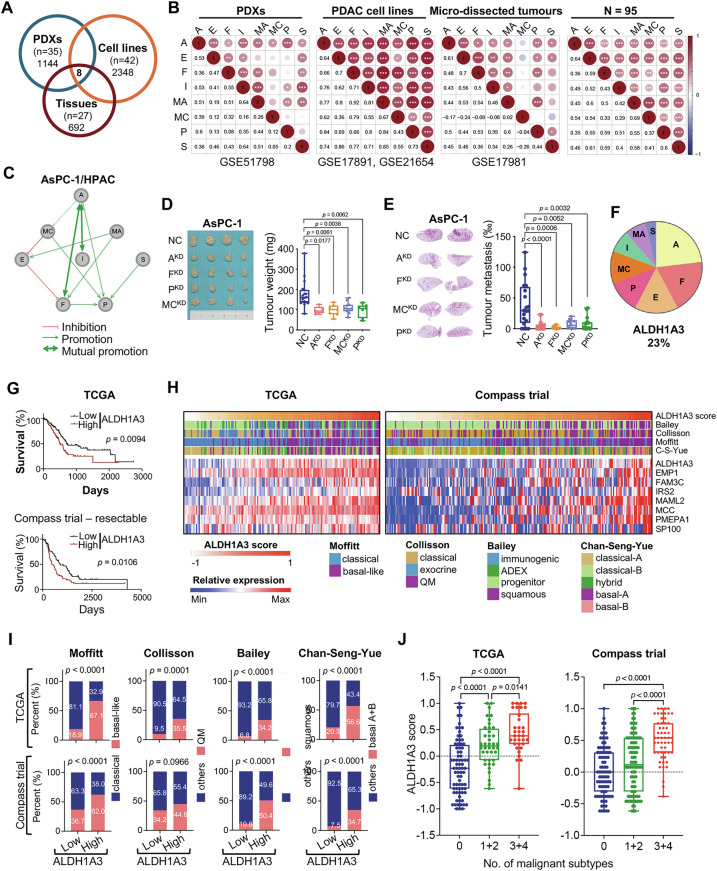
Enriched ALDH1A3-network signature in aggressive PDAC subtypes. **A** Venn diagram showing differentially expressed genes between ALDH1A3-positive and -negative samples in patient-derived xenografts (PDXs), cell lines, and tissues. **B** Spearman´s correlation matrix of 8 genes. ***p < 0.001, **p < 0.01, *p < 0.05. **C** Interaction network analysis depicting relationships among the genes: ALDH1A3 (A), EMP1 (E), FAM3C (F), IRS2 (I), MAML2 (MA), MCC (MC), PMEPA1 (P), and SP100 (S). **D** Subcutaneous xenotransplantation of parental AsPC-1 cells (NC) to immunodeficient mice or genetically manipulated AsPC-1 cells with knockdown (KD) of ALDH13 (A), FAM3C (F), MCC (MC), or PMEPA1 (P), demonstrating effects on tumor 1size and weight. Left: images of tumor xenografts; Right: mean weights and standard deviations. Control (NC, n = 18), ALDH1A3^KD^ (n = 5), FAM3C^KD^ (n = 7), MCC^KD^ (n = 11), PMEPA1^KD^ (n = 7); p values via unpaired Student’s t test. **E** Metastatic lung colonization following tail vein injection in mice; NC (n = 20), ALDH1A3^KD^ (n = 22), FAM3C^KD^ (n = 13), MCC^KD^ (n = 12), PMEPA1^KD^ (n = 15); p values by unpaired t test. **F** Pie chart detailing weight parameters. A (ALDH1A3: 23.08%), F (FAM3C: 19.23%), E (EMP1:15.38%), P (PMEPA1: 11.54%), MC (MCC: 11.54%), I (IRS2: 7.69%), MA (MAML2: 7.69%), S (SP100: 3.85%). **G** Survival analysis for ALDH1A3^High^ versus ALDH1A3^Low^ patients in TCGA and Compass (stage I-III) datasets. **H** Heatmap demonstrating the ALDH1A3 network score and molecular subtype in TCGA and Compass datasets; High-risk PDAC subtypes are highlighted in purple. **I** Proportion of aggressive subtypes versus others in the ALDH1A3^High^ and ALDH1A3^Low^ groups from TCGA and Compass datasets. **J** ALDH1A3 network score in TCGA and Compass datasets, divided by aggressive subtype. *p* values via unpaired t-tests.

We analyzed three microarray datasets from the Gene Expression Omnibus (GEO) database (patient-derived xenografts (PDXs): GSE51798, cell lines: GSE17891 + GSE21654, tissues: GSE17891) to investigate the relationship between ALDH1A3 expression and the aggressive basal-like PDAC subtype [7]. This analysis identified eight key genes (**A**LDH1A3, **E**MP1, **F**AM3C, **I**RS2, **MA**ML2, **MC**C, **P**MEPA1, **S**P100) that distinguish ALDH1A3-positive from negative samples, based on k-means clustering (k = 2, Fig. 1A, Fig. S1A). RNA-seq of samples from 95 patients with PDAC confirmed these results (Fig. 1B). RNA-seq data from TCGA supported these findings and additionally showed an association between the expression of these genes (excluding MCC and IRS2) and lower survival rates in PDAC patients (Fig. S1B). Immunohistochemical (IHC) analysis verified the expression of ALDH1A3, FAM3C, IRS2, MCC, and SP100 in PDAC cells, further validating the association between high ALDH1A3 levels in patient tissues and poor prognosis in a larger cohort of 145 patients (Fig. S1, C, D). However, limited by the semiquantitative nature of IHC, we were not able to establish the correlation between ALDH1A3 expression and these proteins.

To study the interplay among these eight key genetic markers, we used lentiviral shRNA particles to knock down these genes in human AsPC-1 and HPAC PDAC cell lines. We confirmed efficient suppression of these genes at the protein (Fig. S2A) and mRNA levels (Fig. S2B). Functional analysis revealed that the loss of function of any of these genes affected the expression of the others, suggesting potential interactions (Fig. S2C).

Specifically, we observed potential interactions between ALDH1A3 and FAM3C, MCC, PMEPA1, and IRS2. This was demonstrated by constructing an oncogenic network based on transcriptional data (Fig. 1C). Knockdown experiments showed that reducing ALDH1A3 levels decreased the expression of IRS2, FAM3C, MCC, and PMEPA1, and vice versa. These data suggest strong mutual regulation among these genes, as confirmed by western blot analysis (Fig. S2D).

Furthermore, overexpressing ALDH1A3 in ALDH1A3-negative PDAC cells (PANC-1/ALDH1A3^OE^) increased the expression of FAM3C, MCC, and PMEPA1 (Fig. S2E), which was supported by our previous data demonstrating that ALDH1A3 overexpression in PANC-1 cells promoted tumor invasion in vitro [24]. Additionally, cells with knocked down ALDH1A3 or the other key genes FAM3C, PMEPA1, and MCC showed reduced tumor xenograft growth and metastasis in mice, highlighting their role in tumor progression (Fig. 1D, E, Fig. S2, F, G). Knockdown of MAML2 significantly affected cell colony formation in vitro (Fig. S2H), the knockdown of EMP1, IRS2, and SP100 did not affect tumor growth or metastasis (Fig. S2I).

To quantify the impact of individual network genes on the overall network, we developed an ALDH1A3-network score from PDAC mRNA data by assigning numerical weights to each gene based on connectivity (Fig. 1F). Analysis of TCGA (n = 150) and Compass (n = 241) datasets revealed that samples with higher ALDH1A3-network scores correlated with shorter patient survival (Fig. 1G). These scores were also aligned with known unfavorable molecular PDAC subtypes-basal-like, quasi-mesenchymal, squamous, and basal-A/B [2–5] across both datasets (Fig. 1H).

ALDH1A3 mRNA expression was higher in samples from subtypes associated with worse prognosis (Fig. S3A). Specifically, samples identified as unfavorable by three or more classification systems exhibited the highest ALDH1A3-network scores: 85.3% (29/34) in TCGA and 83.3% (45/54) in Compass (Fig. 1I+J). High ALDH1A3-network scores were also prevalent in the glycolytic subtype, which aligns with the role of ALDH1A3 in promoting glycolysis and is indicative of poor prognosis (Fig. S3B–D) [25]. To maintain clarity, we focused on the function of ALDH1A3 in differentiating between classical-like and basal-like PDAC subtypes.

### ALDH1A3 enhances AP-1 activity through FOS family members

**Fig. 2:**
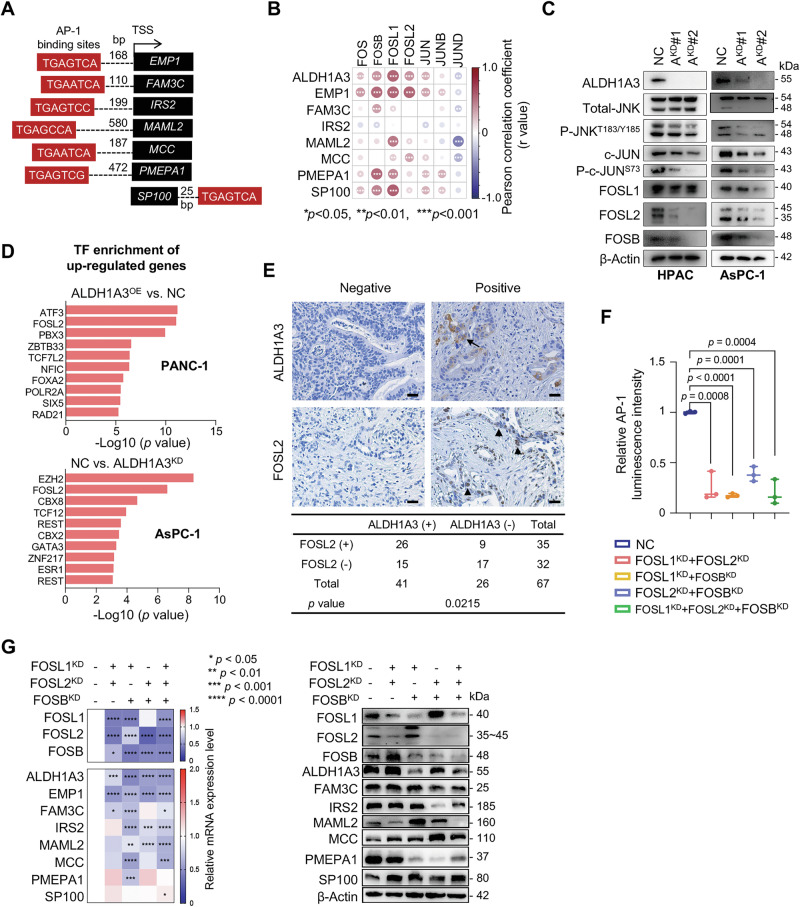
ALDH1A3 regulates AP-1 activity through the FOS family. **A** Screening results for AP-1 binding sites in the promoters of candidate genes. **B** Heatmap illustrating Pearson correlation coefficients between candidate genes and AP-1 subunits from the FOS and JUN families. ***p < 0.001, **p < 0.01, *p < 0.05. **C** Western blot analysis showing protein levels before (NC) and after ALDH1A3 knockdown (A^KD^#1; A^KD^#2) in HPAC and AsPC-1 cells. Key proteins detected include JNK activity markers (p-JNK^T183/Y185^; p-c-JUN^S73^) as well as expression of FOS subunits (FOSB, FOSL1, FOSL2). This panel shows one representative experiment out of three conducted. **D** TF enrichment analysis in PANC-1 and AsPC-1 cells affected by altered ALDH1A3 expression. Analysis based on RNA-seq data from three biological replicates. **E** Contingency table analysis for co-expression of ALDH1A3 and FOSL2 in PDAC sections; statistical significance assessed by Chi-square (χ2) test). IHC images displaying FOSL2/ALDH1A3 staining in PDAC sections, scale bars represent 50 μm. **F** Results from AP-1 luciferase reporter assays in cells subjected to dual or triple knockdown of FOS subunits. Data are presented as mean values from three independent experiments: p values calculated via unpaired t test. **G** RT-qPCR (left) and western blot analysis (right) performed on AsPC-1 cells before and after knockdown of multiple FOS subunits, examining the expression of the previously described 8 candidate genes. One representative result from three independent experiments is displayed.

In silico analysis identified conserved AP-1 binding sites in the promoters of the network genes, except for ALDH1A3 (Fig. 2A). Examination of the TCGA dataset revealed a strong correlation between the expression of ALDH1A3 and AP-1 subunits belonging to the JUN and FOS families (Fig. 2B). Following ALDH1A3 knockdown, there was a notable decrease in the expression of FOSL2 and FOSB, phosphorylation of JUN and activity of c-Jun N-terminal kinase (JNK) in the AsPC-1 and HPAC PDAC cell lines (Fig. 2C, Fig. S4A).

TF enrichment analysis of AsPC-1 cells with ALDH1A3 knockdown and PANC-1 cells with ALDH1A3 overexpression revealed ATF3 and FOSL2 as the top enriched AP-1 related candidates (Fig. 2D). Immunohistochemical analysis of PDAC sections showed a positive correlation between ALDH1A3 and FOSL2 staining, but not with FOSL1 or FOSB (Fig. 2E, Fig. S4B, C), suggesting that ALDH1A3 predominantly modulates AP-1 activity via FOSL2. In support of this, a luciferase reporter assay showed that both knockdown of endogenous ALDH1A3 and overexpression of exogenous ALDH1A3 significantly affected AP-1 activity in the AsPC-1 and PANC-1 cell lines, respectively (Fig. S4D).

Subsequent experiments involving the knockdown of FOSB, FOSL1, or FOSL2 in AsPC-1 and HPAC cells demonstrated that single gene knockdown marginally affected AP-1 activity and the expression of other ALDH1A3 network genes (Fig. S4E–G). However, knockdown of one FOS gene often resulted in the upregulation of other FOS proteins, indicating a possible compensatory mechanism. Only the combined knockdown of two or more FOS proteins significantly reduced AP-1 activity and decreased expression of ALDH1A3 network components, as evidenced by AP-1 luciferase reporter assays, RT-qPCR, and western blot analyses (Fig. 2F–H, Fig. S4H).

### Aldh1a3 is crucial for Jnk/Ap-1 activation and pancreatic carcinogenesis in vivo

**Fig. 3:**
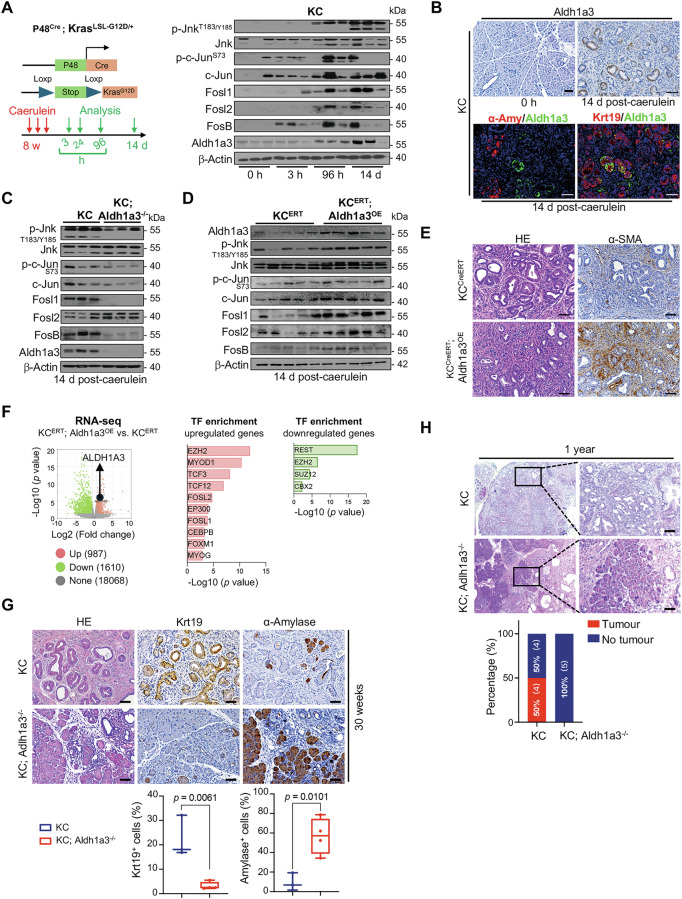
Aldh1a3 is crucial for Jnk/AP-1 activation and pancreatic carcinogenesis in vivo. **A** Scheme of treatment of KC mice with cerulein. Western blot analysis of pancreata from KC mice 0, 3, 96 h (h) and 14 days (d) after cerulein treatment and detection of Jnk activity (p-Jnk^T183/Y185^, p-c-Jun^S73^), expression of Fos subunits (Fosb, Fosl1, Fosl2), and Aldh1a3 ; n = 3/group. **B** IHC displaying Aldh1a3 expression in KC pancreata 14 days after cerulein treatment. Immunofluorescence revealing Aldh1a3/α-amylase and Aldh1a3/Krt19 co-staining; scale bar: 50 μm; n = 3. **C** KC mice and KC; Aldh1a3^-/-^ underwent cerulein treatment and subsequent analysis of protein expression in their pancreas by western blot, as described above. n = 3. **D** Pancreas proteins analyzed by western blot post-cerulein treatment, n = 5. **E** H&E- and α-SMA-stained sections from KC^ERT^; Aldh1a3^OE^ or KC^ERT^ control mice 14 days post-treatment; scale bars: 50 μm, n = 5/group. **F** Differential gene expression analyzed in RNA-seq between KC^ERT^; Aldh1a3^OE^ (n = 3) and KC^ERT^ pancreata, (n = 4). Among 987 upregulated genes the top ten enriched TFs were shown. Among 1, 610 downregulated genes five enriched TFs were shown. (G) H&E-stained pancreas sections depicting KC and KC; Aldh1a3^–/–^ pancreata at 30 weeks. IHC showing Krt19 and α-amylase-positive cells; scale bars: 50 μm, n = 3 (KC), n = 4 (KC; Aldh1a3^–/–^). p values by unpaired t test. **H** IHC results of H&E, Krt19, or α-amylase-stained pancreas sections from one-year-old KC and KC; Aldh1a3^–/–^ mice. Scale bars: 50 μm, n = 5/genotype.

To investigate the correlation between Aldh1a3 expression and Jnk/Ap-1 activation in pancreatic carcinogenesis, we treated KC (p48^Cre/+^; LSL-Kras^G12D/+^) mice with caerulein to induce pancreatitis and Jnk/Ap-1 activity as previously described [26, 27]. Pancreatic tissues collected at various time points after treatment were analyzed by western blotting (Fig. 3A). Following caerulein treatment, we observed coordinated expression of Aldh1a3 and activated Jnk, Fosl1, Fosl2, and Fosb (Fig. 3A, Fig. S5A). In addition, Aldh1a3 was predominantly found in neoplastic lesions of KC pancreata but not in acinar cells, as confirmed by immunohistochemistry and immunofluorescence staining (Fig. 3B, Fig. S5B, C).

To explore a causal relationship between Aldh1a3 expression and Jnk/Ap-1 activation, we used KC mice with a targeted deletion of Aldh1a3 (p48^Cre/+^; LSL-Kras^G12D/+^; Aldh1a3^flox/flox^, referred to as “KC; Aldh1a3^–/–^”, Fig. S5D, E). Compared to parental KC mice, Aldh1a3 deletion resulted in reduced expression of Fosb and Fosl1 and decreased Jnk pathway activation, although Fosl2 protein levels remained unchanged (Fig. 3C, Fig. S5F). To confirm these findings, we generated a transgenic mouse line overexpressing Aldh1a3 (LSL-Rosa^CAG-Aldh1a3^) by introducing loxP-STOP-loxP Aldh1a3 cDNA into the Rosa26 locus. Upon tamoxifen-induced removal of the STOP cassette by p48^CreERT^, exogenous Aldh1a3 was expressed under the control of the CAG promoter in pancreatic acinar cells (Aldh1a3^OE^), followed by caerulein treatment for 2 days (Fig. S6A). As expected, Aldh1a3 overexpression led to increased expression of Fosl1 and Fosb and enhanced Jnk pathway activity (Fig. 3D, Fig. S6B, C), correlating with carcinoma in situ formation and a significant stromal reaction compared to control mice (Fig. 3E, Fig. S6D). RNA-seq of KC^ERT^; Aldh1a3^OE^ (n = 3) versus KC^ERT^ mice (n = 3) revealed 2,597 differentially expressed genes, with Fosl1 and Fosl2 among the top enriched TFs associated with Aldh1a3-mediated upregulated genes (Fig. 3F). Despite these changes, neither loss nor overexpression of Aldh1a3 alone had any apparent physiological effects based on histological analysis of exocrine markers (keratin 19, α-amylase or muc5ac) (fig. S5E, S6D).

Long-term studies revealed that aging KC; Aldh1a3^–/–^ mice (n = 20) for up to one year showed fewer acinar-do-ductal metaplasia (ADM) and intraepithelial neoplasia (PanIN) lesions at 20 and 30 weeks compared to controls, with no invasive PDAC development at one year (0/5), unlike KC mice, where 50% (4/8) developed invasive PDAC (Fig. 3H, I, Fig. S7A, B).

In conclusion, the obtained evidence cross-species underscores that Aldh1a3 is pivotal in promoting Ap-1 activity and PDAC progression.

### ALDH1A3 promotes basal-like transcription by AP-1-mediated enhancer activity

**Fig. 4:**
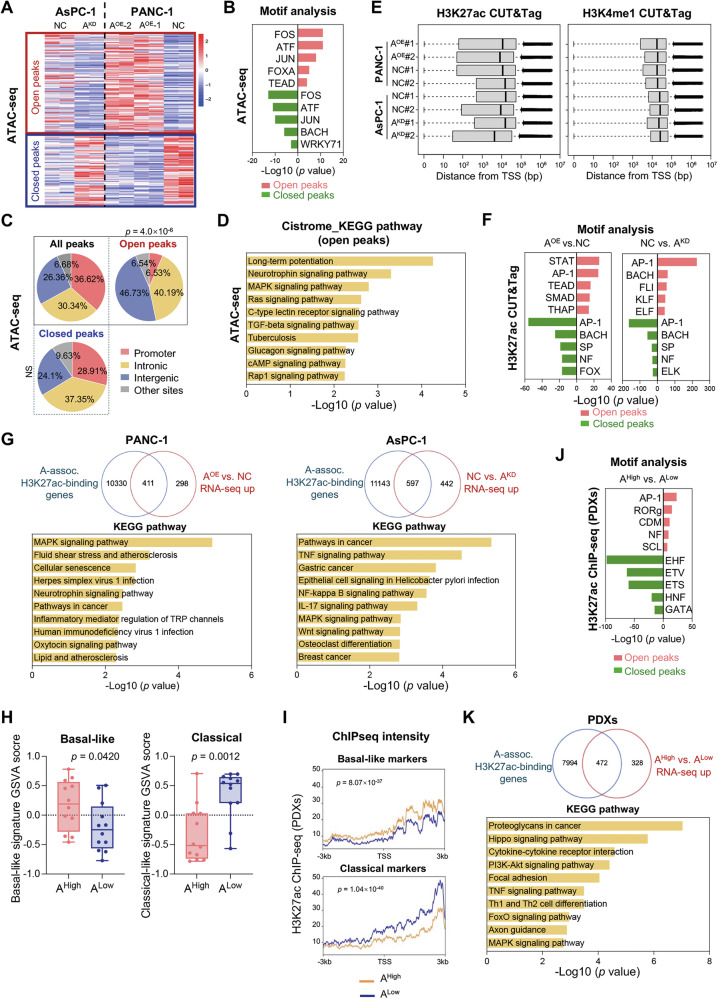
ALDH1A3 promotes an oncogenic, basal-like specific transcriptional program by regulating AP-1-mediated enhancer activity. **A** Heatmap showing differential ATAC-seq results that identify ALDH1A3-associated accessible or inaccessible chromatin regions in PANC-1 and AsPC-1 cells. **B** Identification of the top five TF motifs found at ALDH1A3-accessible or -inaccessible chromatin sites in AsPC-1 and PANC-1 cells, analyzed with n = 2 biological replicates. **C** Pie charts illustrating the genomic distribution of ATAC-seq peaks associated with ALDH1A3, highlighting enrichment of intergenic and intronic sites within open peaks, *p* = 4.0×10^-6^, chi-squared test. **D** KEGG pathway analysis of genes corresponding to ALDH1A3 open peaks derived from ATAC-seq data. **E** Quality control analysis of H3K4me1 and H3K27ac CUT&Tag profiles in PANC-1 and AsPC-1 cells, detailing the distribution of all peaks relative to transcription start sites (TSS) in base pairs (bp), with n = 2 biological replicates. **F** Identification of the top five enriched TF motifs in H3K27ac CUT&Tag data comparing ALDH1A3^OE^ overexpressing and control PANC-1 cells, as well as ALDH1A3^KD^ knockdown versus control AsPC-1 cells, with n = 2 biological replicates. **G** Visualization of overlapping data showing upregulated genes alongside H3K27ac-associated peaks in specific cellular comparisons; accompanied by KEGG pathway analysis, with n = 2 biological replicates in each group. **H** GSVA displaying expression profiles of basal-like and classical markers in 22 patient-derived xenografs (PDXs) with high (ALDH1A3^High^) and low (ALDH1A3^Low^) ALDH1A3 expression. **I** H3K27ac ChIP-seq intensity profiles for basal-like and classical markers across ALDH1A3^High^ and ALDH1A3^Low^ PDXs groups. **J** Top five enriched TF motifs identified in H3K27ac CUT&Tag profiles comparing ALDH1A3^High^ versus ALDH1A3^Low^ PDXs groups. **K** Pie chart showing RNA-seq-based upregulated gene overlap with ALDH1A3-associated H3K27ac peaks in ChIP-seq data between ALDH1A3^High^ versus ALDH1A3^Low^ groups, including KEGG pathway analysis.

To explore the relationship among ALDH1A3 expression, AP-1 activity, and basal-like differentiation, we performed an Assay for Transposase-Accessible Chromatin using sequencing (ATAC-seq) to profile global chromatin accessibility. Over 50,000 genomic sites were analyzed, revealing differential chromatin accessibility at 8781 genomic loci in PANC-1/ALDH1A3^OE^ cells (compared to PANC-1/NC cells) and 2763 genomic loci in AsPC-1/ALDH1A3^KD^ cells (compared to AsPC-1/NC cells). We identified 190 loci with differential accessibility associated with ALDH1A3 expression in both cell types (Fig. 4A). Specifically, PDAC cells expressing ALDH1A3 exhibited increased accessibility in 107 genomic regions and decreased accessibility in 83 genomic regions. TF motif enrichment analysis of these 190 genomic sites using Hypergeometric Optimization of Motif EnRichment (HOMER) highlighted enrichment for AP-1 subunits, particularly the FOS family (Fig. 4B). The 107 regions with increased accessibility (“ALDH1A3-associated open peaks”) were mainly distal intergenic and intronic, indicating enhanced functionality (Fig. 4C, p = 4.0 × 10^6^). Functional annotation of these ALDH1A3-associated open sites using Cistrome-GO predicted functions of cis-regulatory regions (http://go.cistrome.org), showing significant enrichment in “MAPK signaling,” “RAS signaling,” and “TGF-beta signaling” pathways, which are known to promote PDAC and basal-like differentiation (Fig. 4D).

Given the enhancer characteristics of genomic sites with differential chromatin accessibility upon ALDH1A3 expression, we used the Cleavage Under Targets and Tagmentation (CUT&Tag) assay to map H3K27ac (Histone H3 lysine 27 acetylation) and H3K4me1 (H3K4 monomethylation) in PDAC cell lines, histone markers for active and poised gene enhancer sites [28]. The analysis identified 17,998/16,975 ALDH1A3-associated open/closed H3K27ac peaks, and 24,142/46,627 ALDH1A3-associated open/closed H3K4me1 peaks in PANC-1/ALDH1A3^OE^ cells (compared with PANC-1/NC cells). Similarly, we found 22,329/16,773 ALDH1A3-associated open/closed H3K27ac peaks and 27,149/17,020 ALDH1A3-associated open/closed H3K4me1 peaks in AsPC-1/NC cells (compared to AsPC-1/ALDH1A3^KD^ cells). Quality control analysis showed H3K27ac peaks at a median distance between 10^3 ^bp and 10^5 ^bp from the transcription start site (TSS) and H3K4me1 peaks at 10^4 ^bp to 10^5 ^bp, typical for enhancers (Fig. 4E). HOMER motif analysis consistently identified AP-1 consensus motifs as the most significant of these ALDH1A3-associated enhancer regions (Fig. 4F). Additionally, motif analysis identified other PDAC-related TFs, such as SMADs (SMAD2, SAMD3), TEADs (TEAD1, TEAD3), and STATs (STAT5, STAT4, STAT6), which are linked to the basal-like differentiation of PDAC [29].

To identify the gene networks regulated by ALDH1A3-associated enhancers, we integrated RNA-seq and CUT&Tag data for H3K27ac. In PANC-1/ALDH1A3^OE^ cells, 58.0% (411/709) of the upregulated genes had increased H3K27ac peaks (compared to PANC-1/NC), and 57.5% (597/1039) of the upregulated genes in AsPC-1/NC cells exhibited H3K27ac open peaks (compared to AsPC-1/ALDH1A3^KD^ cells, Fig. 4G). KEGG pathway analysis of these genes identified “MAPK signaling,” and “TNF signaling,” pathways as relevant pathways promoting basal-like differentiation of PDAC. Similar results were obtained when an integrated analysis of RNA-seq and CUT&Tag data of H3K4me1 was performed (Fig. S8A, B).

Western blot analysis showed that ALDH1A3 knockdown slightly reduced H3K27ac levels in PDAC cell lines (AsPC-1 and HPAC); however, it had no detectable impact on H3K4me1 levels (Fig. S9A). To assess whether ALDH1A3 influences A-CoA biosynthesis-previously reported to be regulated by ALDH1A3 and involved in histone acetylation [9]-we measured nuclear A-CoA levels using ELISA assays in control and ALDH1A3^KD^ PDAC cell lines. No significant differences were observed (Fig. S9B).

To validate these findings in vivo, we analyzed publicly available Chromatin Immunoprecipitation DNA-Sequencing (ChIP-seq) data for H3K27ac (E-MTAB-5632) and paired transcriptional data (E-MTAB-5639) from 22 patient-derived xenograft (PDX) PDAC tumors [28]. We classified samples into ALDH1A3^High^ and ALDH1A3^Low^ based on the median ALDH1A3 network score. By comparing the transcriptional profiles, we found 1,408 differentially expressed genes (Fig. S8C). We further observed that ALDH1A3^High^ PDX tumors were enriched for basal-like markers, whereas ALDH1A3^Low^ PDX tumors were enriched for classical markers by GSVA [3], consistent with previous observations in human PDAC tissues (Fig. 4H). Comparison of H3K27ac peaks between ALDH1A3^High^ and ALDH1A3^Low^ PDX tumors revealed higher signals at basal-like gene loci in ALDH1A3^High^ tumors, with summits near 3 kb from TSS, and consistently higher signals at classical gene loci in ALDH1A3^Low^ tumors (Fig. 4I). Differential comparison via DESeq2 identified 22,129 H3K27ac peaks (targeting 8,466 genes) more represented in ALDH1A3^High^ PDX tumors, and 18,395 H3K27ac peaks (targeting 8, 356 genes) more represented in ALDH1A3^Low^ PDX tumors. Homer’s motif analysis identified AP-1 as the most significant TF for ALDH1A3^High^ tumors, while GATAs (e.g., GATA4, GATA6) and HNFs (e.g., HNF4A, HNF1B) were significant for ALDH1A3^Low^ tumors (Fig. 4J). Notably, 59% (472/800) of genes elevated in ALDH1A3^High^ tumors had H3K27ac open peaks (Fig. 4K). KEGG pathway analysis highlighted “MAPK signaling,” “TNF signaling,” and “Proteoglycans in cancer” as relevant PDAC pathways. Similar findings were obtained using ChIP-seq data for H3K4me1 (Fig. S8D–F).

Taken together, ALDH1A3 promotes an oncogenic, basal-like transcriptional program by regulating AP-1-mediated enhancer activity, converging on the oncogenic MAPK and TNF signaling pathways.

### An AP-1-dependent enhancer network converges on oncogenic MAPK signaling in ALDH1A3^High^ PDAC

**Fig. 5:**
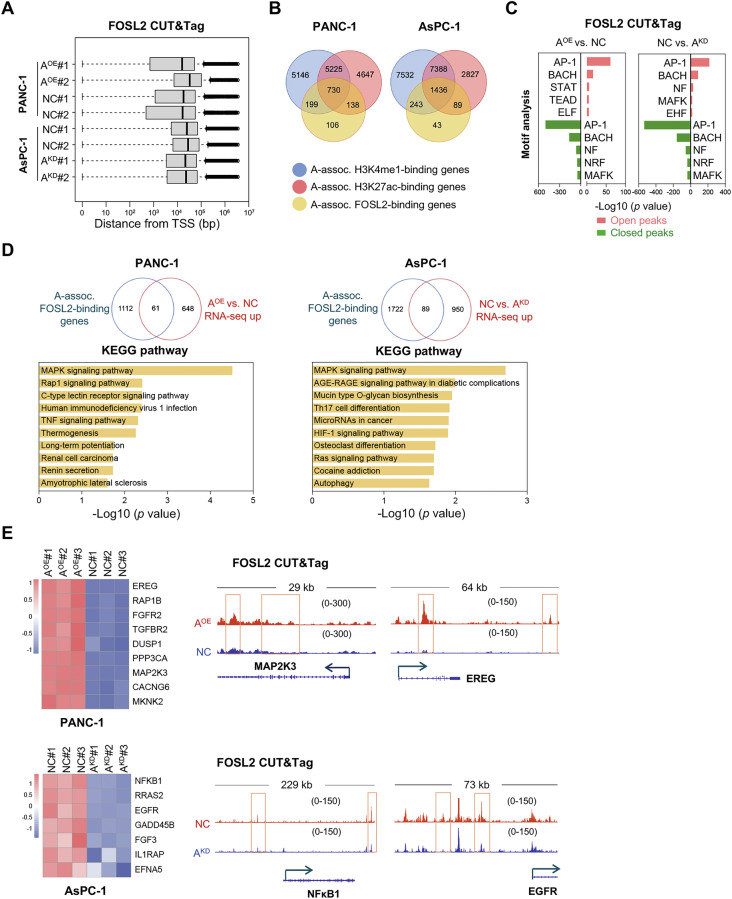
Convergence of the AP-1-dependent enhancer network on the oncogenic MAPK pathway in ALDH1A3^High^ PDAC. **A** Quality control (QC) analysis of FOSL2 CUT&Tag in PANC-1 and AsPC-1 cells, illustrating peak distances from the transcription start side (TSS) in base pairs (bp). Data shown for n = 2 biological replicates. **B** Overlapping pie charts depicting ALDH1A3-associated histone modifications (H3K4me1, H3K27ac) and FOSL2 peaks in PANC-1 cells with ALDH1A3 overexpression (ALDH1A3^OE^) versus control, and AsPC-1cells with ALDH1A3 knockdown (ALDH1A3^KD^) versus control. Analysis conducted with n = 2 biological replicates per group. **C** Top five enriched TF motifs identified in FOSL2 CUT&Tag analysis with open or closed chromatin states in PANC-1/ALDH1A3^OE^ versus PANC-1/control cells and AsPC-1/ control versus AsPC-1/ALDH1A3^KD^ cells. Performed with n = 2 biological replicates. **D** Overlapping charts illustrating upregulated genes co-localized with ALDH1A3-associated FOSL2 peaks across different cell line comparisons including KEGG pathway analysis of these genes. Performed with n = 2 biological replicates. **E** Heat map showing significantly upregulated genes and their association with FOSL2-binding sites linked to the MAPK pathway in PANC-1/ALDH1A3^OE^ versus PANC-1/control cells, and AsPC-1/ control cells versus AsPC-1/ALDH1A3^KD^ cells. IGV tracks showed open chromatin peaks of MAP2K3 and EREG in PANC-1/ALDH1A3^OE^ versus PANC-1/control cells, and NFκB1 and EGFR in AsPC-1/control versus AsPC-1/ALDH1A3^KD^ cells.

Referring to our finding that AP-1, particularly FOSL2, is significantly prevalent in ALDH1A3^High^ PDAC cells and tissues we profiled FOSL2-DNA interactions using the CUT&Tag assay in PANC-1/ALDH1A3^OE^ and AsPC-1/ALDH1A3^KD^ cells. We detected FOSL2 binding peaks at median distance of 10^4 ^bp and 10^5 ^bp from the TSS (Fig. 5A). We detected 1,580 FOSL2-binding sites targeting 1,173 genes in PANC-1 cells (PANC-1/ALDH1A3^OE^ compared to PANC-1/NC) and 2,419 sites in AsPC-1 cells targeting 1,811 genes in AsPC-1/NC compared to AsPC-1/ALDH1A3^KD^. Integrating FOSL2 CUT&Tag data with histone markers (H3K4me1/H3K27ac), we observed a significant overlap of FOSL2-binding genes with active enhancers, specifically 62.2% (730/1, 173) in PANC-1 cells and 79.3% (1, 436/1, 811) in AsPC-1 cells (Fig. 5B). This underscores the critical role of FOSL2 in the enhancement of gene regulation. HOMER motif analysis confirmed the dominant role of AP-1 (Fig. 5C).

Further analysis integrating CUT&Tag and RNA-seq data revealed that 8.6% (61/709) of the genes upregulated in PANC-1/ALDH1A3^OE^ and 8.6% (89/1,039) of the upregulated genes in AsPC-1/NC showed corresponding changes in FOSL2 peaks (Fig. 5D). KEGG pathway analysis indicated that the MAPK, TNF, and HIF-1 signaling pathways are correlated with ALDH1A3^High^ PDAC cells. For instance, mitogen-activated protein kinase kinase 3 (MAP2K3), epiregulin (EREG), epidermal growth factor receptor (EGFR) and nuclear factor kappa B subunit 1 (NF-κB1) are important components of oncogenic MAPK and inflammatory pathways (Fig. 5E).

### RUNX2 is a druggable target in ALDH1A3^High^ PDAC

**Fig. 6:**
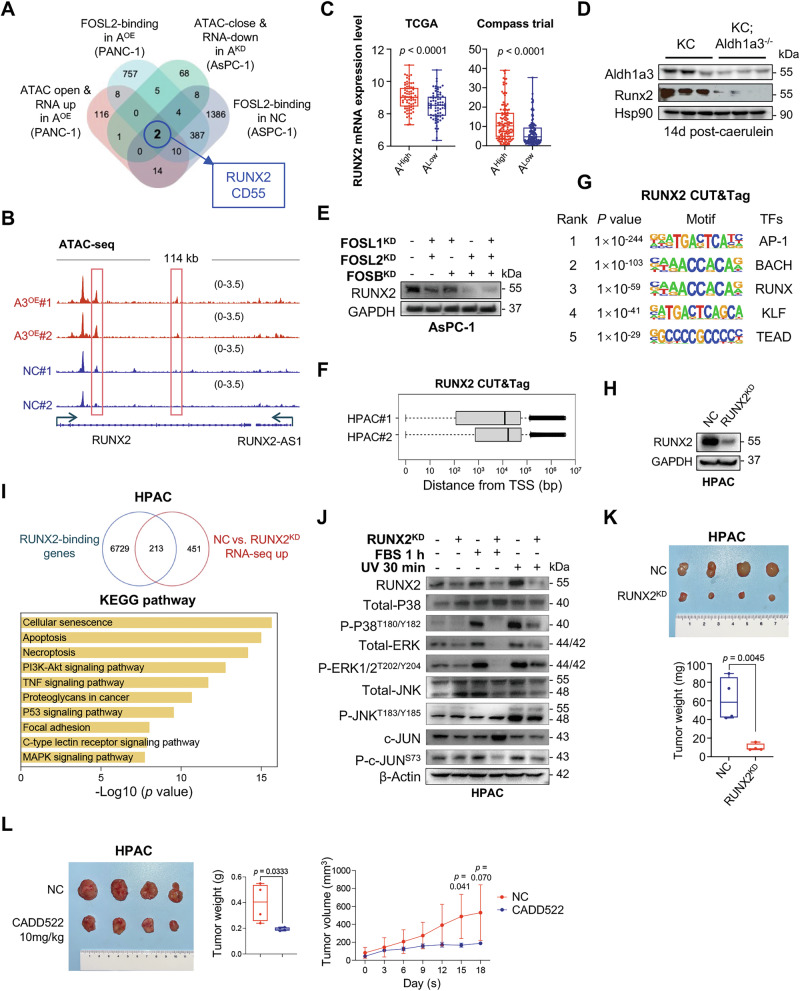
RUNX2 as druggable target of ALDH1A3^High^ PDAC. **A** Integrated analysis of RNA-seq up, ATAC-seq open, and FOSL2 peaks open under ALDH1A3 regulation in PANC-1 and AsPC-1 cells, identifying intersections with RUNX2 and CD55 as shared candidate targets. **B** ATAC-seq data revealing open chromatin sites at the RUNX2 locus in PANC-1 cells overexpressing ALDH1A3 (ALDH1A3^OE^) compared to controls. **C** RUNX2 mRNA expression levels in ALDH1A3^High^ versus ALDH1A3^Low^ groups from TCGA and Compass datasets. **D** Western blot analysis showing RUNX2 protein levels in KC; Aldh1a3^–/–^ pancreata against KC controls, conducted with n = 3 per genotype. **E** Western blot indicating RUNX2 levels post-knockdown of dual or triple FOS subunits in AsPC-1 cells; representative of three similar experiments. **F** Quality control (QC) analysis of RUNX2 CUT&Tag in HPAC cells, showing all peak distances from the transcription start side (TSS) in base pairs (bp). Data from n = 2 biological replicates. **G** Top five enriched TF motifs in RUNX2-binding open chromatin sites in HPAC cells, identified from the RUNX2 CUT&Tag experiment with = 2 biological replicates. **H** Western-blot analysis demonstrating RUNX2 expression in HPAC cells transduced with negative controls (NC) or RUNX2-specific shRNAs; one of three independent experiments is shown. **I** Overlapping charts displaying upregulated genes in HPAC/NC versus HPAC/RUNX2^KD^ and RUNX2-binding genes in HPAC cells, accompanied by KEGG pathway analysis of overlapping genes. Performed with n = 2 biological replicates. **J** Western-blot analysis illustrating activation levels of oncogenic MAPK pathways (p-ERK^T202/Y204^, p-c-JUN^S73^, p-p38^T180/Y182^ and p-JNK^T183/Y185^) and expression of RUNX2 in HPAC/RUNX2^KD^ and control cells treated with FBS for 1 h or irradiated with UV for 30 min, representative of three independent experiments with similar outcome. **K** Xenograft model of HPAC cells demonstrating the effect of RUNX2 knockdown on tumor growth, with n = 4, p values by unpaired Student’s t test. **L** Tumor growth curves, treated with CADD522 (a RUNX2 inhibitor, n = 4) or control (n = 4), in a subcutaneous tumor model generated by HPAC cells; *p* values by unpaired Student’s t test.

To identify potential therapeutic targets in ALDH1A3^High^ PDAC, we combined data from RNA-seq, ATAC-seq, and FOSL2 CUT&Tag assays performed on PDAC cell lines with either overexpressed or knocked down ALDH1A3. RUNX2 and CD55 were identified as strong candidate targets (Fig. 6A, B, Fig. S10A). We focused on RUNX2 because of several compelling findings: (1) TCGA and Compass trial data indicated higher RUNX2 mRNA levels in ALDH1A3^High^ samples than in ALDH1A3^Low^ samples (Fig. 6C); (2) Runx2 expression was lower in pancreata from KC; Aldh1a3^–/–^ mice than in control KC mice (Fig. 6D, Fig. S10B); and (3) RUNX2 was recently reported as a potential marker for basal-like PDAC [29].

Western blot analysis showed the higher expression of RUNX2 in ALHD1A3-positive PDAC cell lines (AsPC-1 and HPAC) and the lower expression of RUNX2 in ALDH1A3-negative PDAC cell line (PANC-1) (Fig. S10C). Furthermore, we found the elevated expression of RUNX2 after overexpressing ALDH1A3 in PANC-1 cell line, and the diminished expression of RUNX2 after knocking down ALDH1A3 in AsPC-1 and HPAC cell lines, respectively (Fig. S10D). Knockdown of FOSL2, along with FOSB or FOSL1, led to a significant decrease in RUNX2 expression, underscoring its regulatory role (Fig. 6E, Fig. S10E). We performed a CUT&Tag assay on the HPAC PDAC cell line, which is high in ALDH1A3, to map RUNX2 binding sites. This assay revealed 10,097 RUNX2 peaks, targeting 6,942 genes, with a median distance of 10^4 ^bp to 10^5 ^bp from the TSS (Fig. 6F). Motif enrichment analysis using Homer highlighted not only AP-1 motifs (FOS, FOSL1, FOSL2), but also BACHs (BACH2, BACH1), RUNXs (RUNX2, RUNX1), KLFs (KLF5, KLF1, KLF6), and TEADs (TEAD3, TEAD1, TEAD4) among the top-ranked (Fig. 6G).

To further investigate the functional impact of RUNX2, we decreased its expression in HPAC cells by lentiviral gene transfer (Fig. 6H). A comparison between HPAC cells with normal controls (NC) and those with RUNX2 knockdown (HPAC/RUNX2^KD^) revealed 664 upregulated genes in the HPAC/NC setup, with 32.1% (213 out of 664) directly regulated by RUNX2, as they had RUNX2 binding sites (Fig. 6I). KEGG enrichment analysis of these genes suggests that the MAPK and TNF signaling pathways were highly enriched.

Further experiments showed that reducing RUNX2 expression diminished JNK, p38, and ERK activities induced by UV light or FBS in HPAC cells, underscoring RUNX2’s crucial role in activating key components of the MAPK pathway (Fig. 6J). When these modified cells were transplanted into immunodeficient mice, RUNX2 suppression significantly slowed down primary tumor growth, demonstrating its therapeutic potential (Fig. 6K). This effect was mirrored in native HPAC cells treated with the RUNX2 inhibitor CADD522 (Fig. 6L). These results are consistent with those of another ALDH1A3^High^ PDAC cell line, AsPC-1 (Fig. S10F–I). In vitro results confirmed RUNX2 inhibitor CADD522 diminished the cell proliferation ability of AsPC-1 and HPAC (Fig. S10J).

## Discussion

Our study elucidated a direct link between high ALDH1A3 expression and the aggressive basal subtype of PDAC, which is associated with poor prognosis. We identified eight genes, including ALDH1A3, and detected a correlation of ALDH1A3-positive samples with decreased survival rates. In vitro knockdown of ALDH1A3 or associated genes such as FAM3C, MCC, PMEPA1, and IRS2 reduced tumor invasion, while in vivo reduction similarly curbed tumor growth and metastasis.

The development of an ALDH1A3-network score was significantly associated with shorter survival and more aggressive PDAC subtypes, underscoring the role of ALDH1A3 in driving an oncogenic basal-like transcriptional program through AP-1-mediated enhancer activity. In line with our findings, ALDH1A3 is known as a metabolic target for cancer diagnosis and therapy across different tumor types [8]. Accordingly, a recent study demonstrated potentiation of transcriptional heterogeneity in melanoma by ALDH1A3-acetaldehyde metabolism [30]. We further explored the current knowledge and suggest a common aggressive cancer mechanism via the FOSL2/AP-1-mediated enhancer network. Our data are consistent with the role of cJUN/AP-1 in specifying basal-like subtypes [14].

We also highlighted the critical involvement of ALDH1A3 in metabolic processes within the nucleus, particularly affecting key metabolites, which impacts histone modification and enhancer landscapes [9], bridging metabolic-epigenetic programming and subtype-specific cellular metabolism. The specific role of ALDH1A3 in the metabolic-epigenetic program fills the gap between the AP-1-mediated enhancer network, subtype specification, and subtype-specific cellular metabolism [25, 31]. Although global H3K27ac levels were indeed reduced following ALDH1A3 knockdown in human PDAC cell lines, no significant changes in nuclear A-CoA levels were observed upon ALDH1A3 modulation. High-quality biochemical studies are therefore warranted to clarify the nuclear metabolites and specific epigenetic enzymes responsible for reprogramming the AP-1-associated enhancer landscape in PDAC cells expressing ALDH1A3. Additionally, the regulatory role of ALDH1A3 on AP-1 activity may be species-specific. While ALDH1A3 expression correlates with FOSL2, but not FOSL1, expression in human PDAC, genetic deletion of Aldh1a3 in the KC mouse model predominantly affected Fosl1 rather than Fosl2 protein levels, adding further complexity to the regulatory relationship between ALDH1A3 and AP-1 activity.

Moreover, our findings imply that RUNX2 is a critical component of the FOSL2/AP-1 enhancer network in PDAC, paralleling its role in osteoblastic differentiation of mesenchymal stem cells [32, 33]. We previously established RUNX2 as a consistent target of TGFβ1 signaling [34], which significantly stimulates the basal-like program in PDAC. Recent studies support this by identifying RUNX2 as a potential marker of basal-like PDAC [29, 35]. In PDAC overexpressing ALDH1A3, RUNX2 is vital within the FOSL2/AP-1 enhancer network, enhancing the transcription of genes critical for oncogenic MAPK and inflammatory TNF signaling pathways. Given these roles, RUNX2 is a promising therapeutic target for PDAC with high ALDH1A3 expression, warranting further exploration in advanced pre-clinical studies.

Although our findings underscore the role of ALDH1A3 in promoting aggressive basal-like PDAC, several limitations merit attention. Primarily, our reliance on cell lines and mouse models may not fully capture the complexities of the human tumor environment. More direct functional assays are needed to delineate the specific effects of ALDH1A3 on PDAC progression. The variability in patient samples and potential biases may also limit the generalizability of our results. Future research should incorporate more diverse patient cohorts and employ advanced models that simulate human PDAC more accurately.

## Supplementary information

Supplementary materials

## Data Availability

All the data underlying the study are available in the manuscript and supplementary files. Additional data supporting the findings of this study will be deposited in public repositories upon acceptance for publication. There are no restrictions on the data availability.
