# Supplementary material for: ALDH1A3 promotes aggressive basal-like pancreatic cancer through an AP-1/RUNX2 enhancer network
Source: Oncogene. 2025 Aug 8;44(40):3774–86. doi: 10.1038/s41388-025-03530-w (PMC12477051; doi:10.1038/s41388-025-03530-w)
Supplement: Supplementary file 1 — Supplementary materials [file 41388_2025_3530_MOESM1_ESM.pdf]

Supplementary Materials for

ALDH1A3 promotes aggressive basal-like pancreatic cancer through an AP-1/RUNX2 enhancer network

Xiaoping Zou *et al.*

*Corresponding authors Email: bo.kong@med.uni-heidelberg.de; shenss@nju.edu.cn

This file includes:

Supplementary methods

Supplementary figure 1-10 and figure legends

Supplementary table 1-4 and table legends

Supplementary methods

Cerulein treatment of mice

The mild acute pancreatitis model was applied to WT (C57BL/6J), KC (Ptf1α$^{Cre/+}$; LSL-Kras$^{G12D/+}$), KC; Aldh1a3$^{-/-}$ (Ptf1α$^{Cre/+}$; LSL-Kras$^{G12D/+}$; Aldh1a3$^{flox/flox}$), KC^{ERT} (Ptf1α$^{CreERTM/+}$; LSL-Kras$^{G12D/+}$) and KC^{ERT}; Aldh1a3^{OE} (Ptf1α$^{CreERTM/+}$; LSL-Kras$^{G12D/+}$; LSL-Rosa$^{CAG\text{-}Aldh1a3}$) animals. The mice, aged 8-9 weeks, were induced by administering cerulein via intraperitoneal (i.p.) injections (0.1 mg/kg body weight, in 100 µl 0.9% NaCl), administered eight times hourly over two consecutive days. Analgesia was provided through subcutaneous administration of Temgesic (0.1 mg/kg body weight) 30 min before and after the first injection every day, and then every 12 h continuously until 72 h after the last injection. The first day of the first injection was considered as day 0, and the time point of the last injection was marked as hour 0. Ultimately, the experimental mice were injected with 2.5 mg of BrdU (5-Bromo-2'-deoxyuridine) two hours before euthanization.

Tamoxifen treatment

Tamoxifen was dissolved in Colza oil containing 10% absolute ethanol. The experimental mice were induced at the age of 5-6 weeks by administering tamoxifen (40 mg/ml, 100 µl per injection) via oral gavage. The administration was conducted every other day, and three applications fulfilled the procedure.

Subcutaneous tumor and lung metastasis models in nude mice

Male BALB/c nu/nu athymic mice, aged 6-8 weeks and referred to as nude mice, were used as subcutaneous tumor and lung metastasis models. For the subcutaneous tumor model, on day 0, cells stably transfected with each gene knockdown were harvested and resuspended at a

concentration of 1×10^8/ml in PBS. A 100 µl cell suspension (containing 1×10^7 cells) was inoculated into the right flank of the mice. The body weight was monitored twice per week for each group. Tumor development was tracked twice weekly using caliper measurements along two orthogonal axes, length (L) and width (W). The volume (V) of the tumor was calculated using the following equation: ($V=L\times W^2/2$). At the time of euthanasia (day 21), blood samples were collected, and tumors were dissected from neighboring connective tissues and weighed. They were then divided into two parts and either frozen in liquid nitrogen or fixed in 4% paraformaldehyde, and embedded in paraffin.

For the lung metastasis model, on day 0, cells stably transfected with each gene knockdown were harvested and resuspended at a concentration of 1×10^7/ml in PBS. A 100 µl cell suspension (containing 1×10^6 cells) was injected into the tail vein of the mice. The body weight was monitored twice per week for each group. Blood samples were collected at the time of euthanasia (Day 21). The right lung (consisting of four lobes) and the left lung (one lobe) were fixed in 4% paraformaldehyde and embedded in paraffin. H&E staining was performed on the largest coronal section, and the metastasis lesion area ratio was calculated and analyzed using Student's t-test.

Immunofluorescence analysis and quantification

Immunofluorescence (IF) was also performed on paraffin-embedded tissue sections. Tissue sections were prepared according to the immunohistochemistry (IHC) protocol. Deparaffination, rehydration, and antigen retrieval were performed in the same manner as for the IHC analysis. The sections were permeabilized with 0.1% PBS-Triton x100 buffer for 10 min. Nonspecific reactivity was blocked with 10% goat serum in PBS (pH 7.4; 0.1 M Tris Base, 1.4 M NaCl). After incubation with primary antibodies overnight at 4 °C, secondary fluorochrome-conjugated antibodies were applied. DAPI staining and mounting were performed simultaneously using the Immunoselect

Antifading Mounting Medium DAPI (Dianova). Sections with complete IF staining were stored at 4 °C. PBST buffer (PBS containing 0.1% Tween 20) was used as the washing buffer throughout the entire procedure. Quantification of IF staining was conducted in a manner similar to that described for the IHC image quantification.

Construction of lentiviral shRNA particles

Lentiviral shRNA particles were provided by GenePharma and GeneChem. The vector hU6-MCS-Ubiquitin-EGFP-IRES-puromycin was used to generate lentiviral shRNA particles. The shRNA-targeting sequence for each gene is available (table S1). PDAC cells were seeded in a 6-well plate at a density of 2×10^5 cells/well. After 24 h, the culture medium was replaced with a fresh media containing 5 μg/ml polybrene. Virus particles were then added at a multiplicity of infection (MOI) of 100 (the ratio of particle number to cell number). After 48 h, the cells infected with the lentivirus were observed to express GFP under a fluorescence microscope. To establish stably transfected cells, cells were continuously cultured with 2 μg/ml puromycin. The cells were regularly monitored under a fluorescence microscope for GFP expression, and the efficiency of gene knockdown was assessed using qRT-PCR.

Generation of PANC-1/ALDH1A3^{OE} cells

The plasmid for the overexpression of ALDH1A3-(ALDH1A3 [NM_000693] Human GFP-Tagged ORF Clone) and the plasmid for the negative control (pCMV6-AC-GFP Tagged Cloning Vector, Origene, PS100010) were purchased from Origene. The plasmids were transfected into PANC-1 cells using Lipofectamine™ 2000 (Thermo Fisher Scientific), according to the manufacturer's instructions. Stably transfected cells were selected using 800 μg/ml G418 (#A1720, Sigma). For the selection of ALDH1A3-overexpression monoclones, stably transfected cells were

seeded in 96-well plates at concentrations ranging from 40 cells/ml to 5 cells/ml. 24 h after seeding, wells containing only one cell were marked and continuously cultured for two weeks. The cells in the marked wells were transferred into larger culture dishes. The expression of ALDH1A3 was tested in each cell clone, and the clone with the highest ALDH1A3 expression was used for further experiments.

RUNX2 inhibition in subcutaneous tumor model

Nude mouse subcutaneous tumor models were established as previously described. Tumor development was tracked twice a week using caliper measurements along two orthogonal axes: length (L) and width (W). The volume (V) of the tumor was calculated using the following equation: ($V = L \times W^2/2$). Once tumors reached 400 mm^3, the mice were randomly divided into four groups, with 3-6 mice in each group. Mice were treated with either the solvent or CADD522 (a RUNX2 inhibitor), at a dose of 10 mg/kg, administered intraperitoneally thrice a week for two weeks.

Immunohistochemistry

IHC was performed using the Dako Envision System (Dako Cytomation GmbH). Consecutive paraffin-embedded tissue sections (3-5 mm thick) were deparaffinized and rehydrated using standard methods. Antigen retrieval was performed by pretreating the slides with citrate buffer (pH 6.0; 10 mM Citric Acid, 0.05% Tween 20) in a microwave oven for 15 min. Endogenous peroxidase activity was quenched by incubation in deionized water containing 3% hydrogen peroxide at room temperature for 10 min. After blocking nonspecific reactivity with TBS (pH 7.4; 0.1 M Tris Base, 1.4 M NaCl) containing 3% BSA, sections were incubated with the respective primary antibody (α S8) at 4 °C overnight. This was followed by incubation with horseradish

peroxidase-linked goat anti-rabbit or mouse antibodies (table S3), a color-reaction with diaminobenzidine, and counterstaining with Mayer's hematoxylin.

mRNA extraction and cDNA preparation, and qRT-PCR

Total RNA was extracted using the TRIzol-based reagent, RNAiso Plus (Takara), according to the manufacturer's instructions. Reverse transcription was performed using the PrimeScript RT Master Mix (Takara). qRT-PCR was performed using a LightCycler 96 system (Roche) and SYBR Green PCR Master Mix (Thermo Fisher Scientific). The target gene expression was normalized to that of the human housekeeping gene ACTB (β-actin). The primer sequences are listed (table S4).

Immunoblot analysis

The cells were washed twice with ice-cold PBS (pH 7.4; 0.01M PBS). Ice-cold modified RIPA buffer containing 1 μg/ml leupeptin and 1 mM PMSF was then added, and cells were homogenized by passing through a G27 syringe needle ten times. The crude homogenate was centrifuged at 14,000 g in a pre-cooled centrifuge for 15 min. The supernatant was immediately transferred to fresh tubes and aliquoted. The protein concentration in the extract was determined using a BCA Protein Assay Kit (Beyotime) according to the manufacturer's instructions. Sample aliquots were either stored at -80 °C or used immediately for western blotting analysis. Total protein (20 to 80 μg) was loaded onto 4~12% polyacrylamide gels and then transferred onto PVDF membranes. Membranes were blocked in 20 ml of Tween-20 (0.05%)-TBS (pH 7.4; 0.1M Tris Base, 1.4M NaCl) containing 5% skimmed milk for 1 hour and then incubated with the respective primary antibody overnight at 4 °C. Membranes were washed three times with 0.05% Tween-20-TBS and then incubated with a horseradish peroxidase (HRP)-conjugated secondary antibody (1:3000) for 1 h at room temperature. Signals were detected using Immobilon ECL Ultra Western HRP

Substrate (Merck Millipore) and were recorded using a 4600 Luminescent Imaging Workstation (Tanon).

AP-1 luciferase reporter assay

The AP-1 luciferase reporter plasmid (pGL4.44[luc2P/AP1 RE/Hygro] Vector) was transfected into pancreatic cancer cells using FuGENE® HD Transfection Reagent (Promega), according to the manual. Twenty-four h after transfection, the cells were treated with molecules accordingly. Six hours after treatment, AP-1 transcription activity was tested using the Dual-Glo® Luciferase Assay System and GloMax®-Multi Detection System (Promega), according to the manufacturer's instructions.

Colony formation assay

The colony formation assay was conducted in 6-well plates. Briefly, the cells were trypsinized and resuspended at a concentration of 150 cells/mL. Three hundred cells per well were seeded in a 6-well plate and cultured for 7-14 days. Colony formation was monitored daily. Once most colonies contained over 50 cells, the cells were fixed with methanol for 20 min and stained with 10% crystal violet. The number of colonies in each well was counted. This experiment was repeated three times and the results were analyzed based on three independent procedures.

RNA-seq

Total RNA was isolated and used for RNA-seq analysis. cDNA library construction and sequencing of PDAC cell lines were performed by the Beijing Genomics Institute using the BGISEQ-500 platform and by Shanghai Jiayin Biotechnology Co., Ltd using the Illumina Nova Seq 6000 platform. cDNA library construction and sequencing of mouse samples and PDCs were

performed by Active Motif Inc. using the Illumina platform. cDNA library construction and sequencing of human PDAC tissues were performed by Shanghai GeneChem Co. Ltd using the HiSeq X ten PE150NovaSeq 6000 platform. High-quality reads were aligned to the human reference genome (hg38) or mouse reference genome (mm10). The expression levels for each gene were normalized to fragments per kilobase of exon model per million mapped reads (FPKM) using RNA-seq by Expectation-Maximization (RSEM). The differential peaks were identified by DESeq2, with thresholds of $|\log2FC| > 0.5$ and $p < 0.05$.

Assay for transposase-accessible chromatin sequencing (ATAC-seq)

ATAC-seq was performed by Jiayin Biotechnology Co., Ltd. and Active Motif Inc. In brief, we collected and spun cells for 5 min at 500 g, 4 °C, and then washed the cells once with 50 µl of cold 1x PBS buffer, which was followed by centrifugation for 5 min at 500 g, 4 °C. Next, the cells were lysed by 50 µl cold lysis buffer (containing 10 mM Tris-HCl, pH 7.4, 10 mM NaCl, 3 mM $MgCl_2$ and 0.1% IGEPAL CA-630) on ice and nuclei were pelleted by centrifugation at 500 g for 10 min. We collected 50,000 nuclei and resuspended them in 50 µl transposase reaction mix from the Nextera DNA Sample Preparation Kit (Illumina) and incubated the reaction at 37 °C for 30 min to perform the Tn-5 transposition reaction. The transposed DNA fragment was purified using a MinElute PCR Purification Kit (Qiagen). Transposed DNA fragments were then amplified using the following PCR conditions: 1 cycle at 72 °C for 5 min and 98 °C for 30 s, followed by 10 cycles at 98 °C for 10 s, 63 °C for 30 s and 2 °C for 2 min. Subsequently, the resulting ATAC-seq libraries were purified using the MinElute Kit (Qiagen), which was 150 bp paired-end sequenced on the Illumina Nova 6000 platform to a depth of 4.0×10^7 reads. Quality distribution plots and base content distributions were generated using FASTQC (http://www.bioinformatics.babraham.ac.uk/projects/fastqc/). Reads with Phred quality scores >

30 were used for further analysis. After removal of the adaptor sequences, the reads were aligned to the hg38 reference genome using the Burrows-Wheeler Aligner (BWA) software. Peaks were called using MACS2 (v2.1.2) software with a cutoff q-value < 0.05. MACS2 analysis generated two types of peaks, narrow and broad. In this study, we used narrow peaks for subsequent analysis. We analyzed differentially accessible peaks by (1) merging the peak files of each sample using the bedtools software; (2) determining the read counts over the bed for each sample using bedtools multicov; (3) and assessing the differential accessible peak using DESeq2. A region was considered differentially accessible if the absolute value of the |log2FC| was > 1 at a *p* value of < 0.05. Peaks were annotated using the function of the annotatePeak of the ChIPseeker. The results of the annotations were counted, and the distribution results were plotted using the plotAnnoPie function of ChIPseeker. Motif analysis of the peak regions was performed using the HOMER function findMotifsGenome.pl. The input file is the peak file and the genome FASTA file. The DNA sequence was extracted from the peak region widened by both 200 pm upstream and downstream according to the peak file, and the sequence was compared with the Motif database to obtain the motif. Pathway analysis was used to determine the significant pathways of the genes, according to the KEGG database. We used ClusterProfiler to select significant pathways, and the threshold of significance was defined by *p*-value.

Cleavage Under Targets and Tagmentation (CUT&Tag) library construction

CUT&Tag libraries were generated as previously described,[35] using the Hyperactive Universal CUT&Tag Assay Kit (Vazyme). Antibodies against histone H3 (mono methyl K4, #ab8895), histone H3 (acetyl K27 #ab4729, both from Abcam), FOSL2 (#19967), and RUNX2 (#12556, both from Cell Signaling Technology) were used to incubate the bead-bound cells. All CUT&Tag libraries were sequenced on the Illumina Nova 6000 platform, and 150 bp paired-end reads were

generated. CUT&Tag sequencing data were processed as previously described.[36] Paired-end reads were aligned to hg38 using bowtie2 (v2.4.1). MACS2 was used for peak calling. The bigwig file was generated by the bam-coverage command in deepTools with CPM normalization.

We analyzed the typical differential peaks in 3 steps. First, the peak files of each sample were merged using bedtools software. Second, the read counts over the bed were determined for each sample using multicov bedtools. Differentially accessible peaks were assessed using DEGseq. A region was considered differentially accessible if the absolute value of the log2 fold change was 1 at $p < 0.05$. The HOMER findMotifsGenome.pl tool was used for motif analyses. The input file is the peak file and the genome FASTA file. The DNA sequence was extracted according to the peak file, and the sequence was compared with the motif database to obtain the motif.

Integrated analysis of RNA-seq and chromatin-immunoprecipitation followed by sequencing data for 22 patient-derived xenografts

RNA-seq and chromatin-immunoprecipitation followed by sequencing (ChIP-seq) data[19] of 22 patient-derived xenograft (PDX) samples were downloaded from the ArrayExpress (E-MTAB-5639 and E-MTAB-5632, ELIXIR Deposition Databases for Biomolecular Data). For the RNA-seq data, the clean reads were aligned to the reference genome hg38 using STAR. Individual transcript read counts for each sample were produced using HTSeq-count. The ALDH1A3 network score of each sample was calculated, and samples were divided into ALDH1A3^{High} and ALDH1A3^{Low} groups, with the average ALDH1A3 score as the cutoff value. The DESeq2 algorithm was used to filter differentially expressed genes between the ALDH1A3High and ALDH1A3Low groups following significance and FDR analysis under the following criteria: $|\log2FC| > 0.5$ and $p < 0.05$.

For ChIP-seq data, the downloaded fastq files were aligned to the hg38 genome using the BWA program. Peaks were called using the MACS2 callpeak function with the input data as the control. Differential comparisons were analyzed in the same way as the CUT&Tag part described above.

Data and materials availability

Data are available in the public open-access repository. The sequencing data of ATAC-seq, CUT&Tag, RNA-seq data of cell lines, and RNA-seq data of transgenic mice models that support the findings of this study have been deposited in the Sequence Read Archive (SRA) and Gene Sequence Archive (GSA) under the accession codes PRJNA987138 and PRJCA018632. The RNA-seq data of human PDAC tissues reported in this paper have been deposited in the Genome Sequence Archive (Genomics, Proteomics & Bioinformatics 2021) in National Genomics Data Center (Nucleic Acids Res 2022), China National Center for Bioinformation / Beijing Institute of Genomics, Chinese Academy of Sciences (GSA-Human: HRA011000) that are publicly accessible at https://ngdc.cncb.ac.cn/gsa-human.

Nuclear and cytoplasmic protein extraction

Nuclear and cytoplasmic protein extraction was performed using a Nuclear and Cytoplasmic Protein Extraction Kit (Beyotime, Shanghai, China). Briefly, 2×106 cells were resuspended in 200 μl of cytoplasmic protein isolation solution A and homogenized on ice. Then, 10 μl of cytoplasmic protein isolation solution B was added, and the cells were further homogenized on ice. The homogenate was centrifuged at 10 000 × g for 5 min at 4 °C. The resulting supernatant was the cytoplasmic protein fraction. The pellet was resuspended in 50 μl of nuclear protein isolation solution, homogenized on ice and centrifuged at 10 000 × g for 10 min. The resulting supernatant was the nuclear protein fraction.

Quantification of Acetyl Coenzyme A (A-coA) concentration

After nuclear and cytoplasmic protein extraction as described above, the A-coA concentration in the nucleus or cytoplasm was quantified by using the Enzyme-linked Immunosorbent assay (ELISA) kit for A-coA detection (#E-EL-0125c, Elabscience Biotechnology Co.,Ltd, China), following the instructions provided in the commercial manual.

Cell proliferation analysis

HPAC and AsPC-1 cells were plated into 96-well plates at a concentration of $10^{\wedge 3}$ cells per well in 100μL complete growth medium. CADD522 (0, 50 or 100μM) was added to the cells 24 hours after seeding. Cell viability was analyzed 1, 2, 3 and 4 days after cell seeding with Cell Counting Kit-8 (Dojindo, Kumamoto, Japan) according to the manufacturer's instructions.

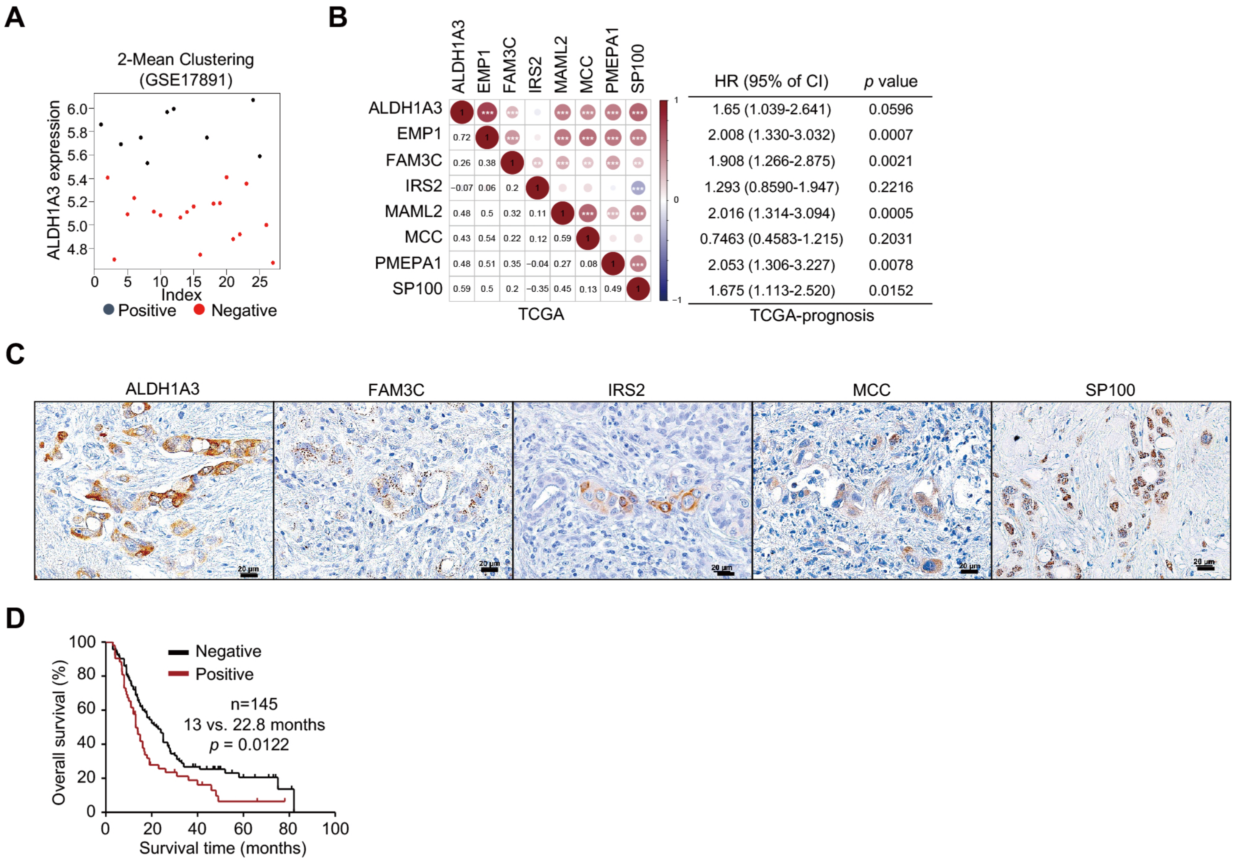

Fig. S1. **(A)** 2-means clustering defines ALDH1A3-positive and negative samples. **(B)** The correlation among 8 genes and the prognostic value of these genes in the TCGA dataset (pearson correlation analysis), ****: $p < 0.0001$, ***: $p < 0.001$, **: $p < 0.01$, * $p < 0.05$. **(C)** Representative IHC images showing cancer cells positive for ALDH1A3, FAM3C, IRS2, MCC, and SP100 in human PDAC tissue sections, scale bar: 20 µm. **(D)** Survival analysis showing that patients with PDAC whose cancer tissues are highly stained for ALDH1A3 survived significantly shorter than those with ALDH1A3 negativity (median survival: 13 vs 22.8 months, log-rank test: $p = 0.0122$). The cancer tissues were obtained from patients prior to surgery (Department of Surgery, Klinikum rechts der Isar, Technical University Munich).

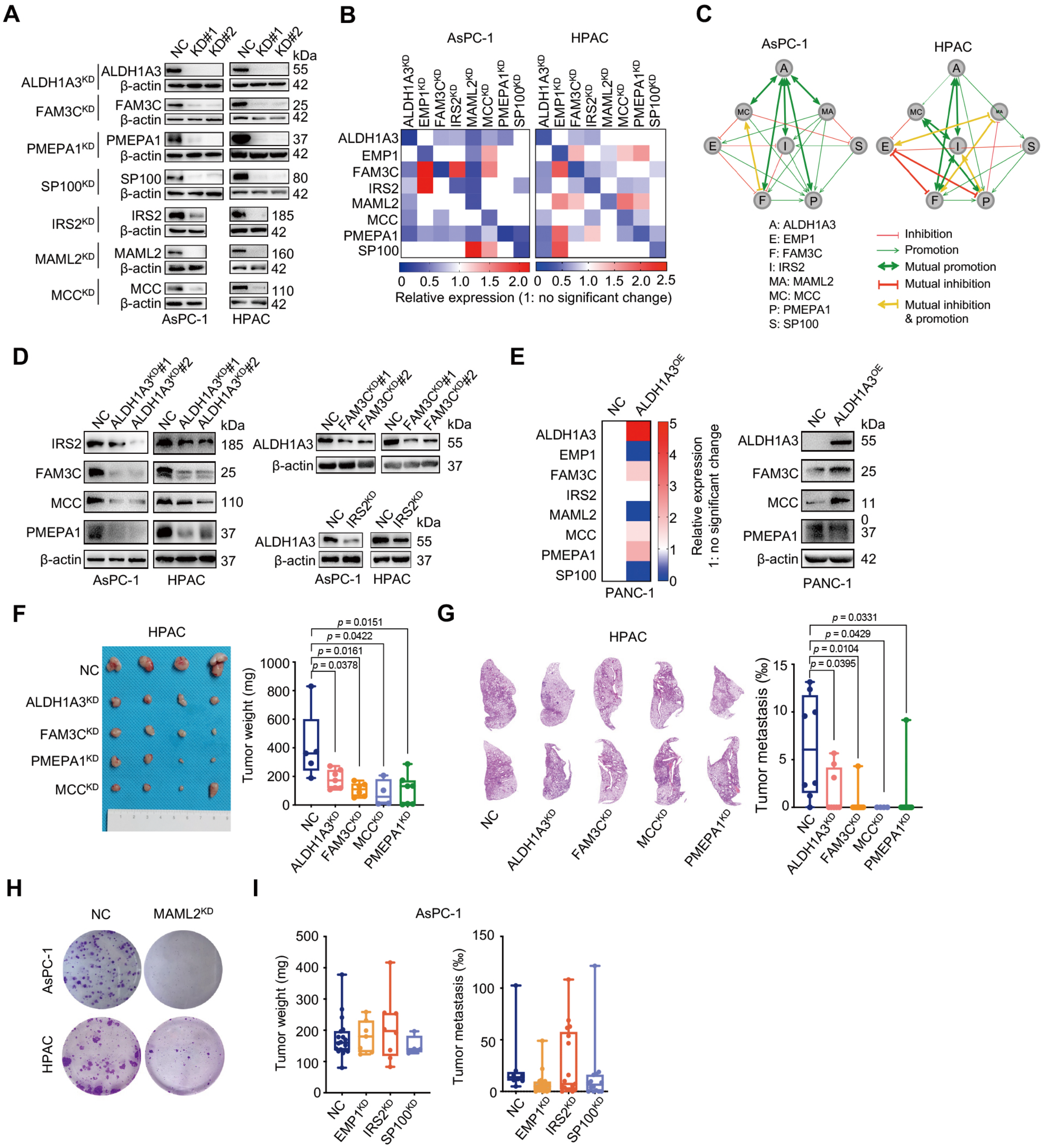

Fig. S2. (A) Western blot analysis showing the levels of ALDH1A3, FAM3C, PMEPA1, SP100, IRS2, MAML2 and MCC in HPAC and AsPC-1 cells after lentiviral shRNA transduction; 1 of 3 independent experiments is shown. **(B)** A heatmap showing dynamic changes in seven other markers after knocking down one component of ALDH1A3 network in AsPC-1 and HPAC cells.

Blocks with white color represent the genes with no significant change. Blue ones show genes' expression levels decreasing significantly, and red ones show those increasing significantly. **(C)** The experimentally validated oncogenic network in AsPC-1 and HPAC cells. **(D)** Western-blot analysis showing the mutual promotions between ALDH1A3 and IRS2/FAM3C/MCC/PMEPA1 in AsPC-1 and HPAC cells; 1 of 3 independent experiments is shown. **(E)** A heatmap showing dynamic changes in seven other markers on the mRNA level after overexpressing ALDH1A3 in PANC-1 cells; western-blot analysis shows the levels of FAM3C, PMEPA1, IRS2, and MCC in PANC-1 cells after overexpressing ALDH1A3; 1 of 3 independent experiments is shown. Blocks with white color represent the genes with no significant change. Blue ones show genes' expression levels decreasing significantly, and red ones show those increasing significantly. **(F)** Subcutaneous tumors generated by negative control cells and ALDH1A3 or FAM3C or MCC PMEAP1-knockdown cells in HPAC cells; NC: negative control; KD: knockdown; NC (n=5), ALDH1A3^{KD} (n=7), FAM3C^{KD} (n=6), MCC^{KD} (n=4) and PMEPA1^{KD} (n=7). *p*-values by unpaired student's t-test. **(G)** Metastatic colonization of the lung generated by negative control cells and ALDH1A3 or FAM3C or MCC PMEAP1-knockdown cells in HPAC cells, and the metastatic area in lung lobes was calculated; NC: negative control; KD: knockdown; NC (n=8), ALDH1A3^{KD} (n=8), FAM3C^{KD} (n=8), MCC^{KD} (n=4) and PMEPA1^{KD} (n=8). *p*-values by unpaired student's t-test. **(H)** The colony-formation assay showing the function of MAML2 on tumor growth in AsPC-1 and HPAC cells; 1 of 3 independent experiments is shown. (**i**) No significant effect of EMP1, IRS2 and SP100 on tumor growth and metastasis in AsPC-1 cells; Tumor growth groups: NC (n=18), EMP1^{KD} (n=7), IRS2^{KD} (n=4) and SP100^{KD} (n=8); Lung metastasis groups: NC (n=8), EMP1^{KD} (n=20), IRS2^{KD} (n=17) and SP100^{KD} (n=14).

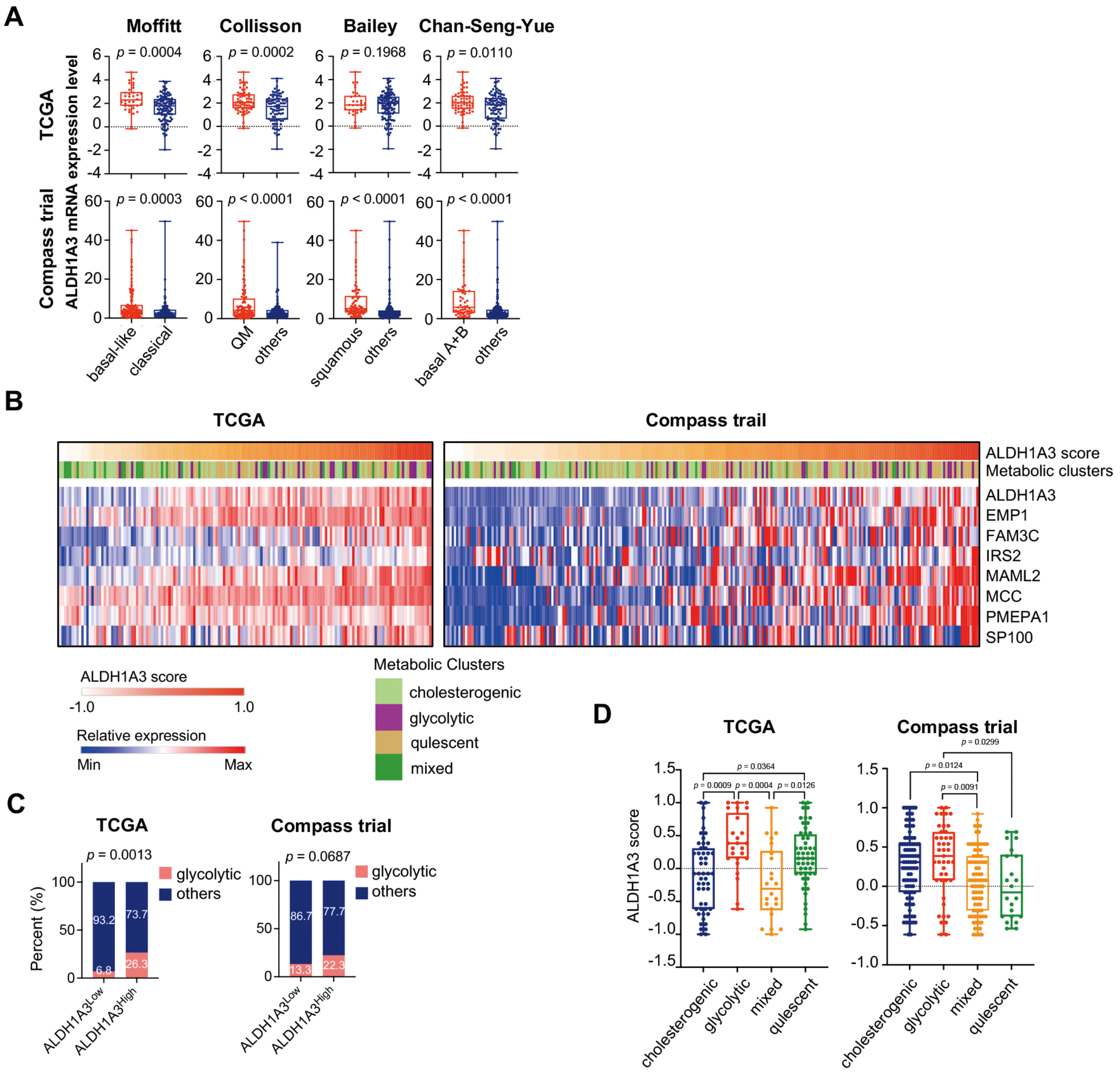

Fig. S3. (A) ALDH1A3 mRNA expression level of each sample belonging to different subtypes in TCGA and Compass datasets. *p*-values by unpaired student's t-test. **(B)** ALDH1A3 network score and the metabolic subtype of each sample in TCGA and Compass trial datasets; PDAC samples were sorted into metabolic subtypes based on the published signatures and ranked by ALDH1A3 network score. **(C)** Proportion of glycolytic and other metabolic subtypes in ALDH1A3^{High} and ALDH1A3^{Low} group in the TCGA and Compass datasets. **(D)** ALDH1A3

network score of each sample belonging to different metabolic subtypes in TCGA and Compass datasets. *p*-values by unpaired student's t-test.

A

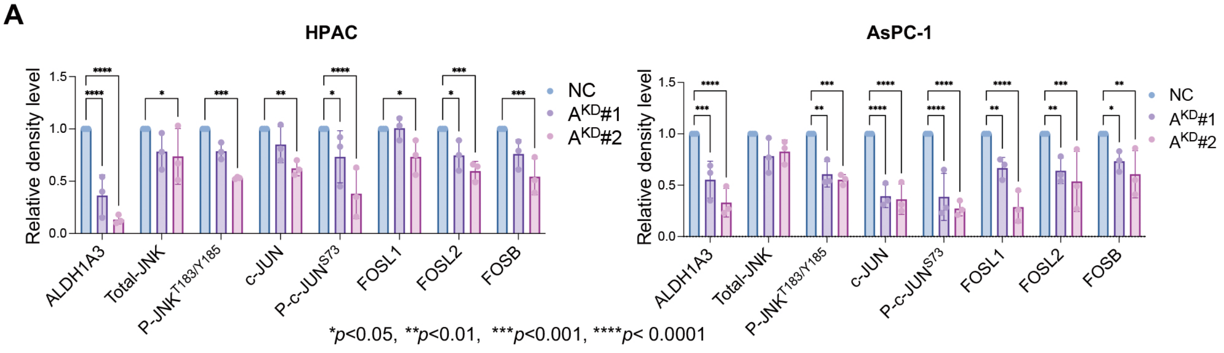

B

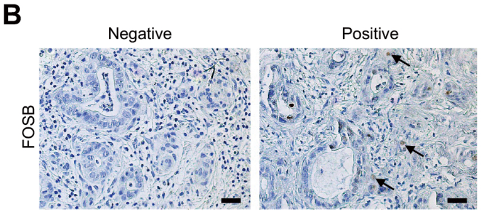

	ALDH1A3 (+)	ALDH1A3 (-)	Total
FOSB (+)	41	20	61
FOSB (-)	5	4	9
Total	46	24	70
p value		0.4916	

C

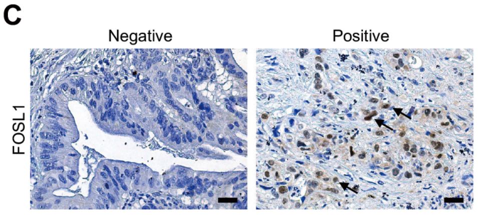

	ALDH1A3 (+)	ALDH1A3 (-)	Total
FOSL1 (+)	30	17	47
FOSL1 (-)	14	11	25
Total	44	28	72
p value		0.5164	

E

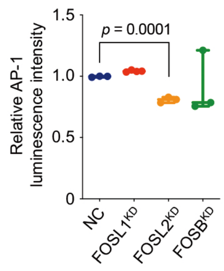

D **F** **G** **H**

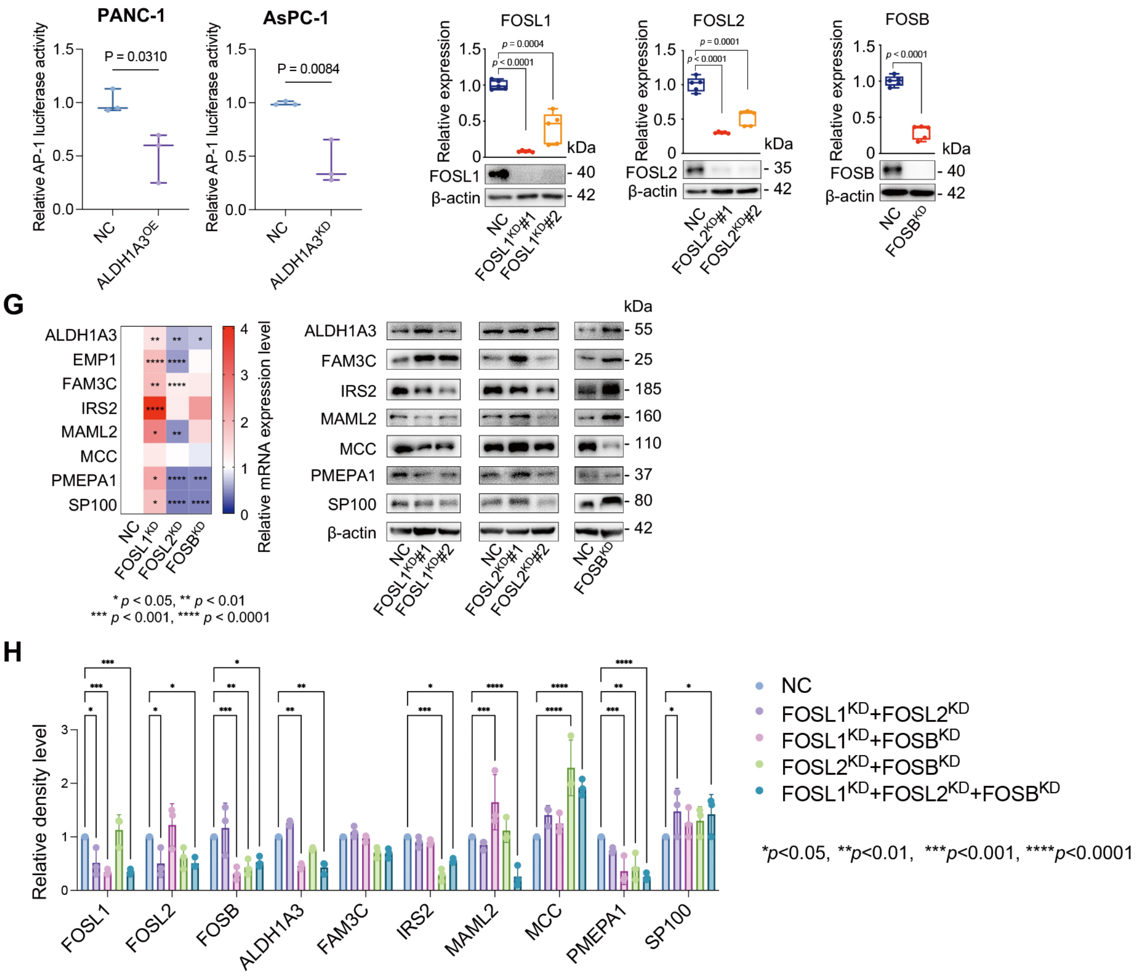

Fig. S4. (A) Relative density levels of western blots in Fig. 2C. *$p<0.05$, **$p<0.01$, ***$p<0.001$, ****$p< 0.0001$. **(B)** Contingency table of ALDH1A3- and FOSB-positive human PDAC sections (*p*-values by Chi-square ($\chi2$) test), representative IHC images demonstrate FOSB-positive and negative staining in human PDAC sections, scale bars: 50 μm. **(C)** Contingency table of ALDH1A3- and FOSL1-positive human PDAC sections (*p*-values by Chi-square ($\chi2$) test), representative IHC images demonstrate FOSL1-positive and negative staining in human PDAC sections, scale bars: 50 μm. **(D)** The Luciferase reporter assays showing AP-1 luciferase activity in AsPC-1 (NC/ALDH1A3^{KD}) and PANC-1 (NC/ALDH1A3^{OE}) cells. **(E)** Knockdown efficiency on mRNA and protein levels for transducing shRNAs specific for FOSB, FOSL1 and FOSL2 in AsPC-1 cells; 1 of 3 independent experiments is shown. **(F)** The luciferase reporter assays demonstrating AP-1 activity in AsPC-1 cells after knocking down FOSL1 (FOSL1^{KD}), or FOSL2 (FOSL2^{KD}), or FOSB (FOSB^{KD}), results of three independent experiments are shown. *p*-values by unpaired student's t-test. **(G)** A heatmap showing the mRNA levels of eight genes upon knocking down FOSL1, or FOSL2 or FOSB. *p*-values by unpaired student's t-test. ****: $p < 0.0001$, ***: $p< 0.001$, **: $p< 0.01$, * $p< 0.05$. Western-blot analysis shows the protein levels of these genes (except for EMP1), 1 of 3 independent experiments is shown. **(H)** Relative density levels of western blots in Fig. 2G. *$p<0.05$, **$p<0.01$, ***$p<0.001$, ****$p< 0.0001$.

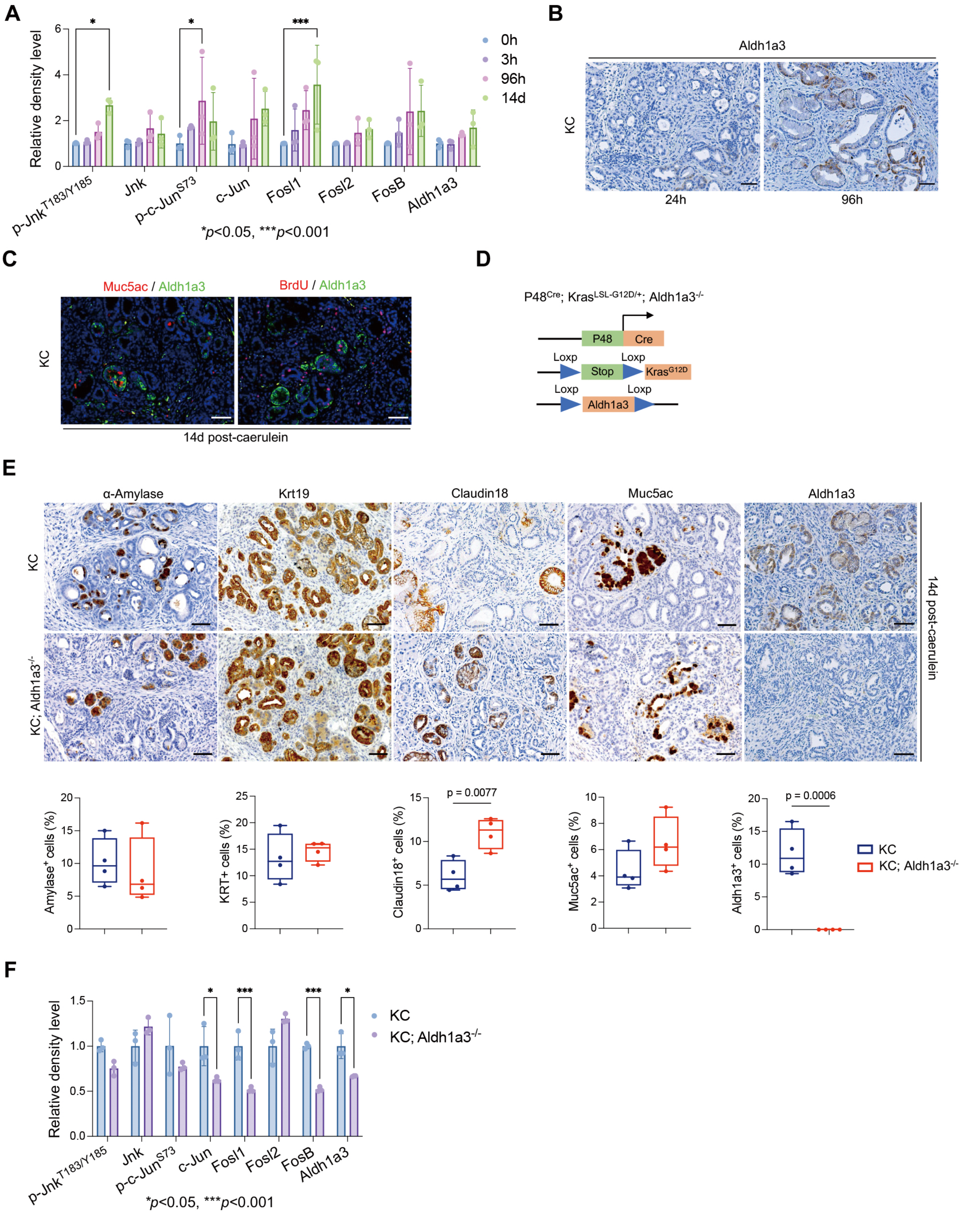

Fig. S5. (A) Relative density levels of western blots in Fig. 3A. **p*<0.05, ***p*<0.01, ****p*<0.001, *****p*< 0.0001. **(B)** IHC images showing Aldh1a3-expressing cells in KC pancreata 24 and 96

hours after caerulein treatment, n=3 (24 h), n=3 (96 h), scale bars: 50 µm. **(C)** Immunofluorescence staining showing pre-neoplastic lesions double-positive for Aldh1a3/Muc5ac or Aldh1A3/BrdU in KC pancreata 14 days after caerulein treatment, n=3 (14 d), scale bars: 50 µm. **(D)** Scheme of KC mice genotype with Aldh1a3 deletion. **(E)** IHC images showing the expression of Aldh1a3, α-amylase, Krt19, Claudin 18, and Muc5ac (Mucin 5 subtype A and C) in in KC and KC; Aldh1a3$^{-/-}$ pancreata 14 days after caerulein treatment, n=3 (KC), n=4 (KC; Aldh1a3$^{-/-}$), scale bars: 50 µm. **(F)** Relative density levels of western blots in Fig. 3C. *p<0.05, **p<0.01, ***p<0.001, ****p< 0.0001.

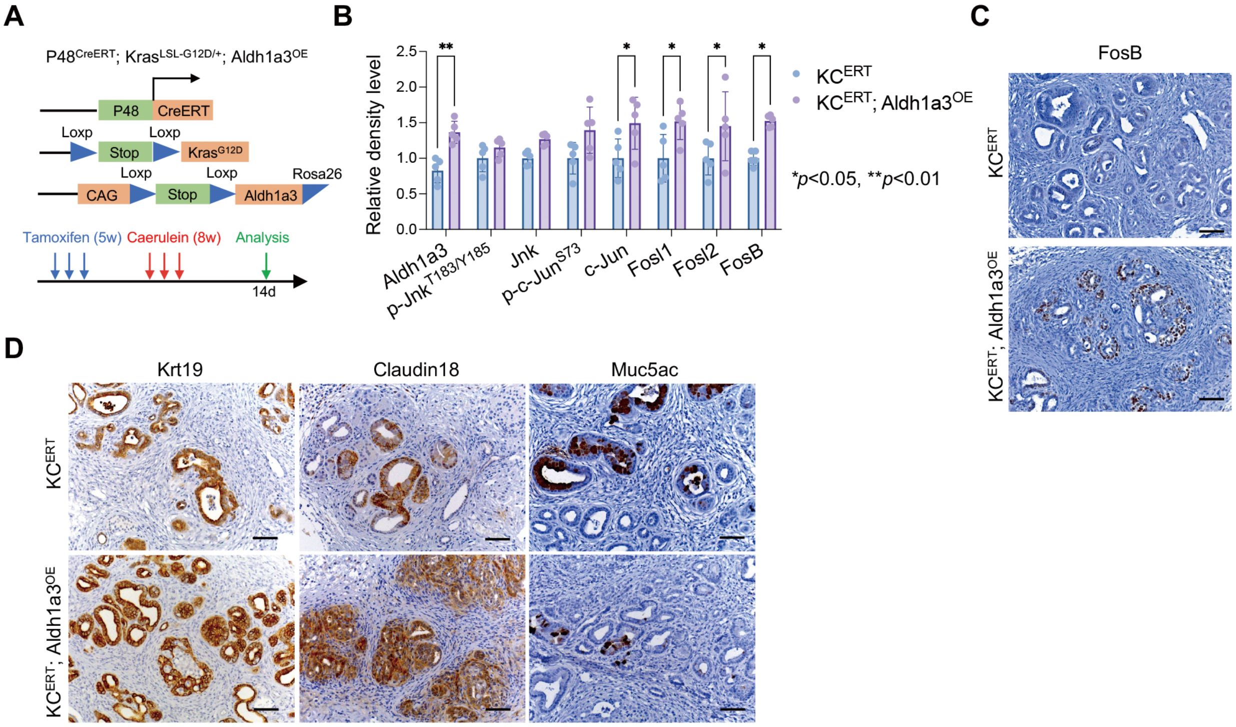

Fig. S6. (A) Diagram of Aldh1a3 overexpressing KC mouse genotype (KC^{ERT}; Aldh1a3^{OE}). **(B)** Relative density levels of western blots in Fig. 3D. *p<0.05, **p<0.01, ***p<0.001, ****p< 0.0001. **(C)** IHC staining demonstrating Fosb-positive cells in KC^{ERT} and KC^{ERT}; Aldh1a3^{OE} pancreata 14 days after caerulein treatment, n=5 (KC^{ERT}), n=5 (KC^{ERT}; Aldh1a3^{OE}), scale bars: 50 µm (**D**) IHC staining demonstrating the Krt19-, Claudin 18-, and Muc5ac-positive lesions in KC^{ERT} and KC^{ERT}; Aldh1a3^{OE} pancreata 14 days after caerulein treatment, n=5 (KC^{ERT}), n=5 (KC^{ERT}; Aldh1a3^{OE}), scale bars: 50 µm.

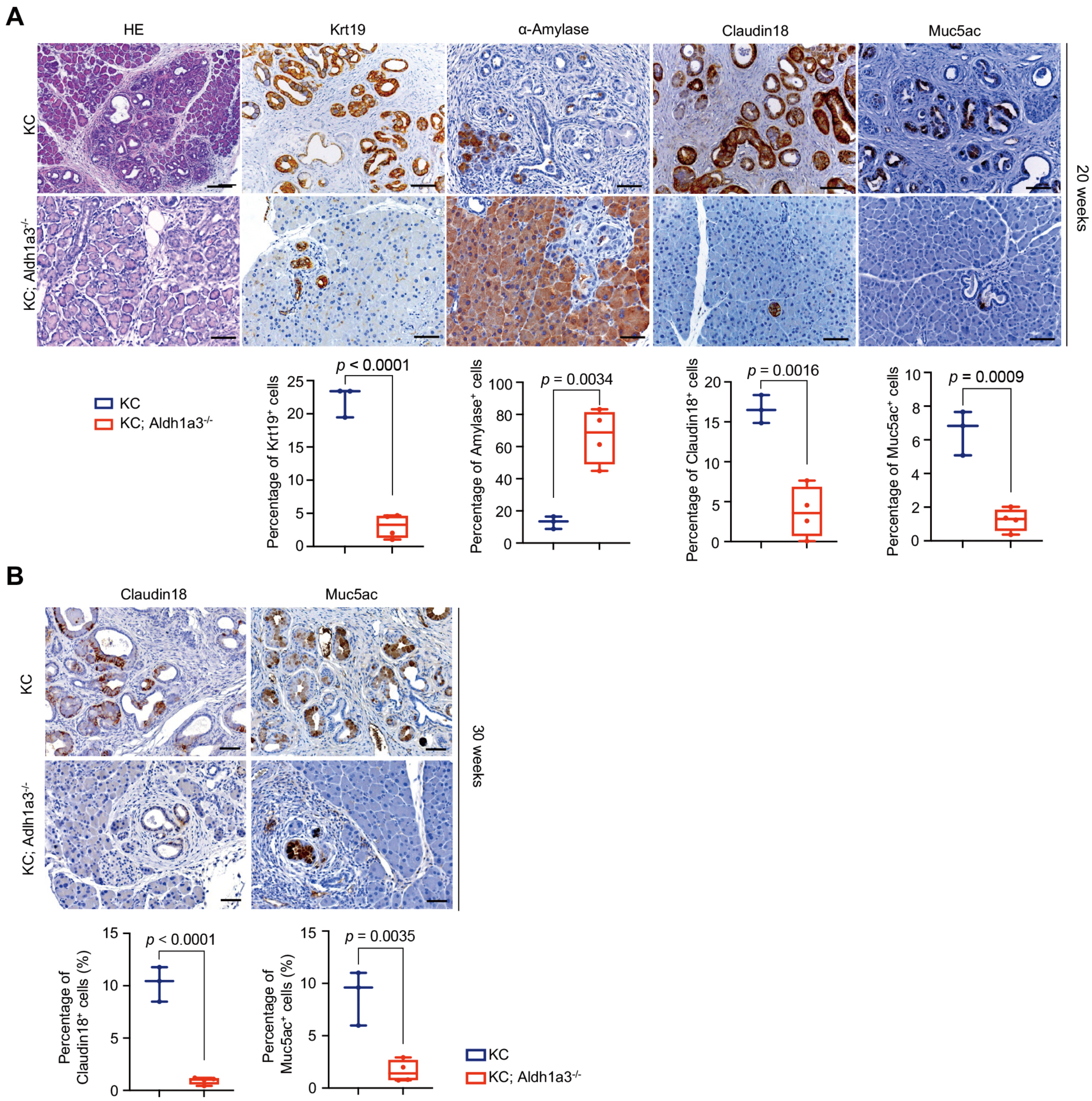

Fig. S7. (A) Representative H&E-stained sections showing the histology of KC and KC; Aldh1a3$^{-/-}$ pancreata collected at 20 weeks of age; representative IHC images demonstrate Krt19-, α-amylase-, Claudin 18- and Muc5ac-positive lesions in these animals, scale bars: 50 µm; n=3 (KC), n=4 (KC; Aldh1a3$^{-/-}$). *p*-values by unpaired student's t-test. **(B)** Representative IHC images demonstrating Claudin18- and Muc5ac-positive lesions in KC and KC; Aldh1a3$^{-/-}$ pancreata

collected at 30 weeks of age, scale bars: 50 μm, n=3 (KC), n=4 (KC; Aldh1a3$^{-/-}$). *p*-values by unpaired student's t-test.

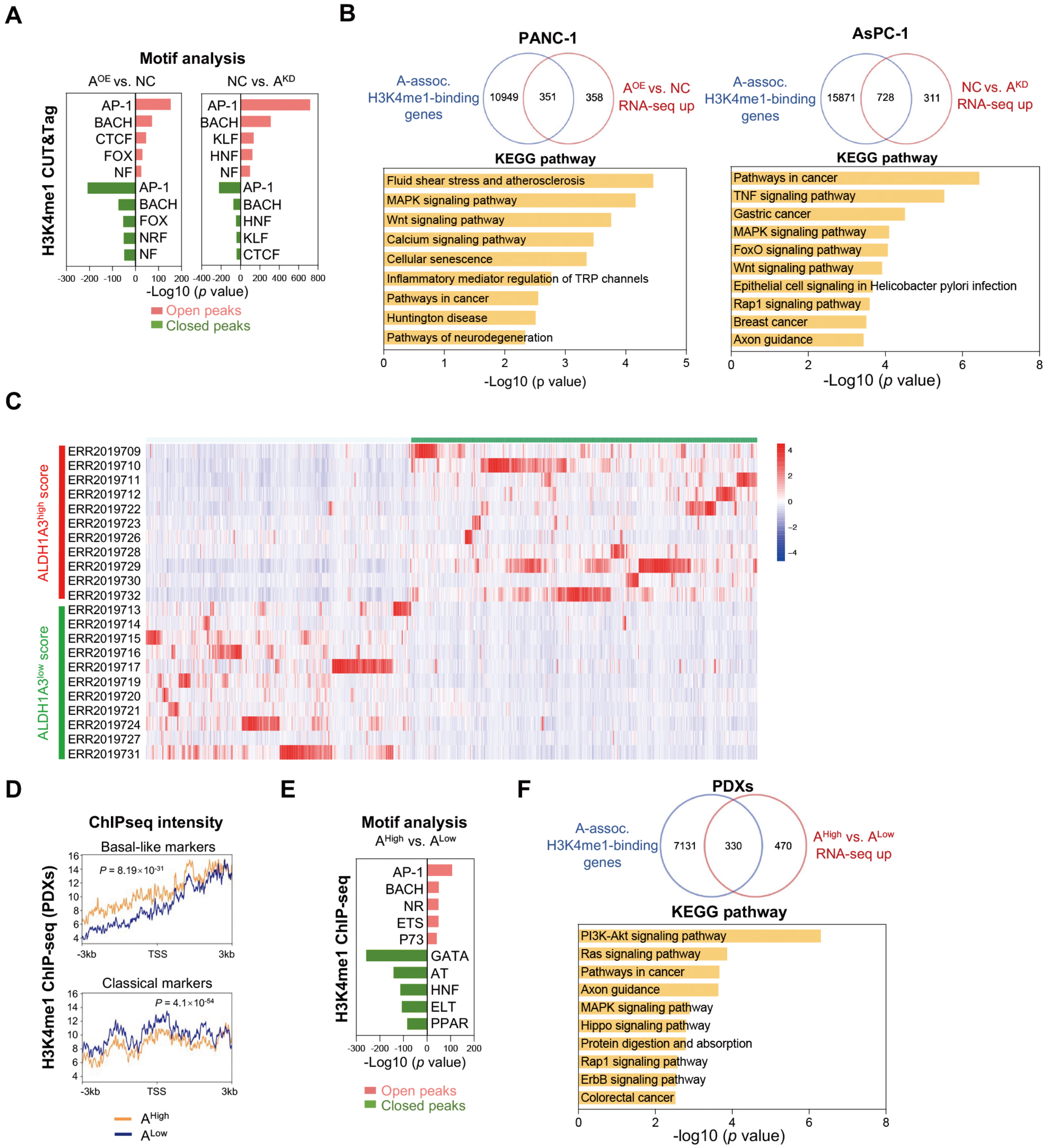

Fig. S8. (A) Top five enriched TF motifs in H3K4me1 CUT&Tag with open or closed peaks are presented in PANC-1/ALDH1A3^{OE} vs. PANC-1/control cells, and AsPC-1/control cells vs. AsPC-1/ALDH1A3^{KD} cells. N = 2 biological replicas in each. **(B)** Overlapping charts displaying up-regulated genes and ALDH1A3-associated H3K4me1-binding peaks in PANC-1, AsPC-1 cell comparisons; KEGG analysis of overlapping genes is displayed. N = 2 biological replicas in each.

(C) A heatmap showing the transcriptional profile of 22 PDXs samples (ALDH1A3^{High} vs. ALDH1A3^{Low} group). (**D**) H3K4me1 ChIP-seq intensity of basal-like or classical markers (Moffitt, et al.) in ALDH1A3^{High} and ALDH1A3^{Low} PDXs groups. (**E**) Top five enriched TF motifs in H3K4me1 CUT&Tag with open or closed peaks are presented in ALDH1A3^{High} vs. ALDH1A3^{Low} PDXs groups. (**F**) The overlap pie chart illustrating the number of up-regulated genes in RNA-seq and ALDH1A3-associated to H3K4me1 peaks in ChIP-seq data in ALDH1A3^{High} vs. ALDH1A3^{Low} groups, KEGG pathway analysis of overlapping genes was performed.

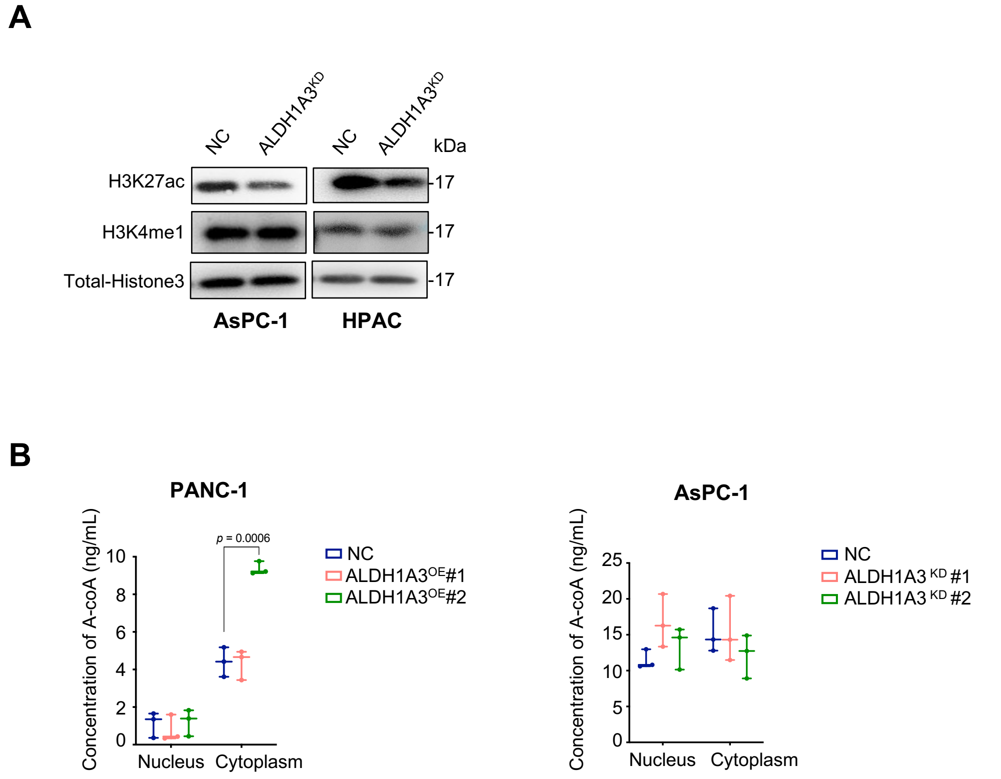

Fig. S9. (A) Western blot analysis showing the levels of H3K27ac and H3K4me1 in HPAC and AsPC-1 cells after lentiviral shRNA transduction of ALDH1A3; 1 of 3 independent experiments is shown. **(B)** Nucleus and cytoplasmic A-CoA concentrations in PANC-1/ALDH1A3^{OE} vs. control cells, and in AsPC-1/ALDH1A3^{KD} vs. control cells were measured by ELISA assay; 1 of 3 independent experiments is shown. p-values by unpaired student's t-test.

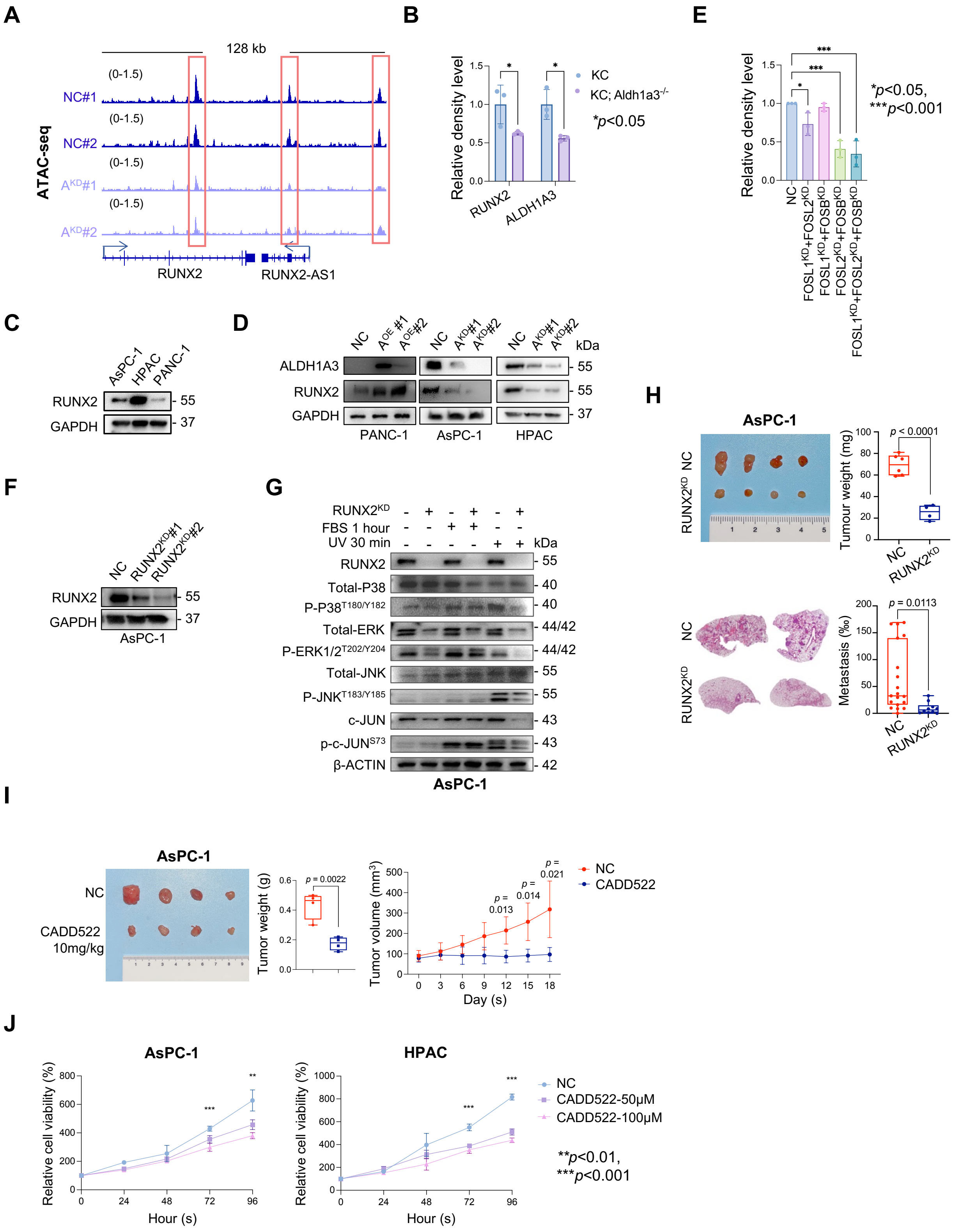

Fig. S10. **(A)** ATAC-seq tracks illustrating the closed sites at RUNX2 loci in AcPC-1/ALDH1A3^{KD} vs. control cells. **(B)** Relative density levels of western blots in Fig. 6D. *p<0.05,

$p<0.01$, ***$p<0.001$, ****$p<0.0001$. **(C) Western-blot analysis showing the basal protein level in AsPC-1, HPAC and PANC-1 cell lines. **(D)** Western-blot analysis showing the protein expression level of RUNX2 and ALDH1A3 in PANC-1/ALDH1A3^{OE}, AsPC-1/ALDH1A3^{KD}, HPAC/ALDH1A3^{KD}, and control cells; 1 of 3 independent experiments is shown. **(E)** Relative density levels of western blots in Fig. 6E. *$p<0.05$, **$p<0.01$, ***$p<0.001$, ****$p<0.0001$. **(F)** Western-blot analysis demonstrating RUNX2 expression in AsPC-1 cells transduced with negative controls (NC) or RUNX2-specific shRNAs; one of three independent experiments is shown**. (G)** Western-blot analysis illustrating activation levels of oncogenic MAPK pathways (p-ERK$^{T202/Y204}$, p-c-JUN^{S73}, p-p38$^{T180/Y182}$ and p-JNK$^{T183/Y185}$) and expression of RUNX2 in AsPC-1/RUNX2^{KD} and control cells treated with FBS for 1 hour or irradiated with UV for 30 min, representative of three independent experiments with similar outcome. **(H)** Xenograft model of AsPC-1 cells showing the effect of RUNX2 knockdown on tumor growth and metastatic colonization, n=4, p-values by unpaired Student's t-test. **(I)** Tumor growth curves, treated with CADD522 (a RUNX2 inhibitor, n=4) or control (n=4), in the subcutaneous tumor model generated by AsPC-1 cells. *p*-values by unpaired student's t-test. **(J)** CCK8 assay showed relative cell viability of HPAC and AsPC-1 cells treated with negative control, CADD522 (50, 100μM) for 0, 24, 48 and 72 hours.

Table S1. List of shRNA sequences

Gene	ShRNA 1# (5'-3')	ShRNA 2# (5'-3')
ALDH1A3	GAGCAGGTCTACTCTGAGTTT	GAGCGAATAGCACCGACTATG
EMP1	CAGTATCACCACGGCTATT	CTACTGTTATTATGCTATT
FAM3C	CATACAAGATGGAACAATA	TGCAAGTTTAGGAAATCTA
IRS2	TGGATGAATACACCCTGAT	
MAML2	TGTTTAACATGGGCTTAAA	
MCC	AAGCAAGATTAGAGAGTTT	
PMEPA1	GTTTGTTCAGATCATCATC	CCCTATGAATTGTACGTTT
SP100	GTACAATGTTCTTAGTGAA	AGGCAGAGCTACACAACCA
Scramble	TTCTCCGAACGTGTCACGT	
RUNX2	TACCTATCACAGAGCAATTAA	
FOSL1	GGATGGTACAGCCTCATTTCC	GGAGACTGACAAACTGGAAGA
FOSL2	GGATTATCCCGGGAACTTTGA	GCTCTGTCATCAAGCCCATCA
FOSB	GAAGGAACGTCTGGAGTTTGT	

Table S2. List of primary antibodies

Antibody Name	Catalog Number	Application* (reactivity**)	Manufacturer
Rabbit anti-ALDH1A3	HPA046271	WB; IHC (H)	Sigma-Aldrich
Rabbit anti-FAM3C	ab72182	WB (H); IHC	Abcam
Rabbit anti-IRS2	3089	WB (H); IHC	Cell signalling Technology
Rabbit anti-MAML2	6988	WB (H);	Cell signalling Technology
Rabbit anti-MCC	HPA037390	WB (H); IHC	Sigma-Aldrich
Mouse anti-TMEPAI	H00056937-M01	WB (H)	Abnova
Rabbit anti-SP100	HPA016707	WB (H); IHC	Sigma-Aldrich
p44/42 MAPK (Erk1/2)	9102	WB (H)	Cell signalling Technology
Phospho-p44/42 MAPK (Erk1/2) (Thr202/Tyr204)	9101	WB (H)	Cell signalling Technology
p38 MAPK	9212	WB (H)	Cell signalling Technology
Phospho-p38 MAPK (Thr180/Tyr182)	4511	WB (H)	Cell signalling Technology
SAPK/JNK	9252	WB (H)	Cell signalling Technology
Phospho-SAPK/JNK (Thr183/Tyr185)	9251	WB (H)	Cell signalling Technology
c-Jun rabbit mAb	9165	WB (H)	Cell signalling Technology
Phospho-c-Jun (Ser73)	3270	WB (H)	Cell signalling Technology
Mouse anti-Fra1	sc-376148	WB; IHC (H)	Santa Cruz Biotechnology
Rabbit anti-Fra2	19967	WB; IHC (H); ChIP	Cell signalling Technology
Rabbit anti-FosB	2251	WB; IHC (H)	Cell signalling Technology
Mouse anti-BrdU	5292	IHC (H)	Cell signalling Technology
Mouse anti-β-Actin	sc-69879	WB (H)	Santa Cruz Biotechnology
Mouse anti-GAPDH	TA802519	WB (H)	Origene
Rabbit anti-β Tubulin	ab6046	WB (H)	Abcam
Rabbit anti-RUNX2	12556	WB (H, M)	Cell signalling Technology
Anti-Histone H3 (mono methyl K4) antibody	ab8895	ChIP	Abcam
Anti-Histone H3 (acetyl K27) antibody	ab4729	ChIP	Abcam
Histone H3 (D1H2) XP® Rabbit mAb	4499	WB (H)	Cell signalling Technology

Table S3. List of secondary antibodies

**Antibody name**	**Catalog number**	**Application* (reactivity**)**	**Producer**
Goat anti-Mouse Immunoglobulins/HRP	P0447	IHC (H)	Dako
Goat anti-Rabbit Immunoglobulins/HRP	P0448	IHC (H)	Dako
Goat anti-rabbit IgG, HRP-linked Antibody	7074	WB (H)	Cell signalling Technology
Horse anti-mouse IgG, HRP-linked Antibody	7076	WB (H)	Cell signalling Technology

Table S4. List of primer sequences for qRT-PCR analysis of human genes

**Gene Name**	**Forward (5'-3')**	**Reverse (5'-3')**
ALDH1A3	ACTCTGAGTTTGTCAGGCGG	CACTGGCCCGAAAATCTCCT
EMP1	AGATGCCCTCAAGACAGTGC	TGGACACCCCCACAAGAATG
FAM3C	AACGTGGTGGGACCCAAAAT	CATCTCCTCCCCACATGTCA
IRS2	GGCATTCCAGCCCCTATGTT	GAGAACTGCCACACACTGGT
MAML2	TGGACATGACTCGGTGCAAT	TCACCCCCGGCTCTATTCTAA
MCC	CCCCACATTCACACTGATGC	AGGGGCTCTTACTCACTTGC
PMEPA1	TTATCTCCCCTGCAAAGCCC	GACGACCAATGAGGACAGGG
SP100	CACTGACGTTGATGAGCCCT	AATCTGGGGTCGTGAGCAAG
ACTB	CTACGTCGCCCTGGACTTCGAGC	GATGGAGCCGCCGATCCACACGG
FOSB	CTGGCGGAGGTGAGAGATTT	TTAACAACGGGGAAGGGGTC
FOSL1	AACCCTCCTCGCTTTGTGAG	GAAACAGTGGGCAGCTTTGG
FOSL2	GCCCAGTGTGCAAGATTAGC	GGGCTCCTGTTTCACCACTA
